# ADAM8 signaling drives neutrophil migration and ARDS severity

**DOI:** 10.1172/jci.insight.149870

**Published:** 2022-02-08

**Authors:** Catharina Conrad, Daniela Yildiz, Simon J. Cleary, Andreas Margraf, Lena Cook, Uwe Schlomann, Barry Panaretou, Jessica L. Bowser, Harry Karmouty-Quintana, Jiwen Li, Nathaniel K. Berg, Samuel C. Martin, Ahmad Aljohmani, S. Farshid Moussavi-Harami, Kristin M. Wang, Jennifer J. Tian, Mélia Magnen, Colin Valet, Longhui Qiu, Jonathan P. Singer, Holger K. Eltzschig, CAPSys Study Group, Wilhelm Bertrams, Susanne Herold, Norbert Suttorp, Bernd Schmeck, Zachary T. Ball, Alexander Zarbock, Mark R. Looney, Jörg W. Bartsch

**Affiliations:** 1 Department of Medicine, Division of Pulmonary, Critical Care, Allergy and Sleep Medicine, School of Medicine, University of California, San Francisco, San Francisco, California, USA.; 2 Department of Anesthesiology, Intensive Care and Pain Medicine, University Hospital Münster, Münster, Germany.; 3 Institute of Experimental and Clinical Pharmacology and Toxicology, PZMS, ZHMB, Saarland University, Homburg, Germany.; 4 Department of Neurosurgery/Lab, Faculty of Medicine, Philipps-University, Marburg, Germany.; 5 School of Cancer & Pharmaceutical Sciences, Faculty of Life Sciences & Medicine, King’s College London, London, United Kingdom.; 6 Department of Pathology & Laboratory Medicine, School of Medicine, University of North Carolina, Chapel Hill, North Carolina, USA.; 7 Department of Biochemistry and Molecular Biology, and; 8 Department of Anesthesiology, McGovern Medical School at The University of Texas Health Science Center at Houston, Houston, Texas, USA.; 9 Department of Chemistry, Rice University, Houston, Texas, USA.; 10 Department of Pediatrics, Division of Pediatric Critical Care, University of California, San Francisco, San Francisco, California, USA.; 11 See Supplemental Acknowledgments for CAPSys Study Group details.; 12 Institute for Lung Research (iLung), Philipps-University, Marburg, Germany.; 13 Department of Internal Medicine II, University Medical Center Giessen and Marburg, Giessen, Germany.; 14 Deutsches Zentrum für Lungenforschung (DZL), Giessen, Germany.; 15 Department of Internal Medicine/Infectious Diseases and Respiratory Medicine, Charité – Universitätsmedizin Berlin, Berlin, Germany.; 16 Pulmonary and Critical Care Medicine, University Medical Center Giessen and Marburg, Marburg, Germany.; 17 German Center for Infectious Disease Research (DZIF), Marburg, Germany.; 18 Center for Synthetic Microbiology (SYNMIKRO), Marburg, Germany.

**Keywords:** Inflammation, Pulmonology, Innate immunity, Neutrophils, Proteases

## Abstract

Acute respiratory distress syndrome (ARDS) results in catastrophic lung failure and has an urgent, unmet need for improved early recognition and therapeutic development. Neutrophil influx is a hallmark of ARDS and is associated with the release of tissue-destructive immune effectors, such as matrix metalloproteinases (MMPs) and membrane-anchored metalloproteinase disintegrins (ADAMs). Here, we observed using intravital microscopy that *Adam8^–/–^* mice had impaired neutrophil transmigration. In mouse pneumonia models, both genetic deletion and pharmacologic inhibition of ADAM8 attenuated neutrophil infiltration and lung injury while improving bacterial containment. Unexpectedly, the alterations of neutrophil function were not attributable to impaired proteolysis but resulted from reduced intracellular interactions of ADAM8 with the actin-based motor molecule Myosin1f that suppressed neutrophil motility. In 2 ARDS cohorts, we analyzed lung fluid proteolytic signatures and identified that ADAM8 activity was positively correlated with disease severity. We propose that in acute inflammatory lung diseases such as pneumonia and ARDS, ADAM8 inhibition might allow fine-tuning of neutrophil responses for therapeutic gain.

## Introduction

Acute respiratory distress syndrome (ARDS) is defined as the acute onset of respiratory failure, refractory hypoxemia, and bilateral, noncardiogenic pulmonary edema (1). ARDS represents a stereotypic response to various etiologies and is most commonly a complication of pneumonia or sepsis (2–5). Although advances in supportive care (6–8) and lung-protective mechanical ventilation (9, 10) have improved outcomes, the death rate remains high and ranges from 27% to 46% (2, 9, 11). A major reason for the high mortality rate is that no targeted therapies currently exist. Recently, this unmet need has been highlighted by the large number of patients with ARDS due to the pandemic spread of COVID-19 resulting from SARS-CoV-2 infections (12, 13). Despite considerable research efforts in the past decades, almost all promising therapies discovered in preclinical studies have failed to show clear benefits in clinical trials of ARDS (11, 14–16). A route to overcoming this lack of success may be to develop more specific therapeutic strategies and rapid diagnostic tools to stratify and monitor patients with ARDS in the critical care setting (17).

The pathogenesis of ARDS involves immune-mediated injury of the lung endothelium and epithelium, resulting in alveolar-capillary barrier dysfunction and edema formation (1, 9). Neutrophils are central cellular mediators in both animal and clinical studies of ARDS (18), but all attempts to harness their powerful functions have failed. As first responders of the immune system, neutrophils are armed with several proteases, which enable cell migration and pathogen destruction, but these proteases can also cause damage to the lung when neutrophil recruitment is excessive (19–21). Matrix metalloproteinases (MMPs) and a disintegrin and metalloproteinases (ADAMs) fall within the spectrum of “double-edged” immune modulators and are 2 related groups of enzymes that substantially shape the inflammatory response through extracellular matrix (ECM) degradation and the processing of growth factors, cytokines, and receptors (22, 23). Upon pulmonary insults, proteases are readily activated, and their catalytic activities reflect cellular responses to lung damage (24–26).

The metalloproteinase-disintegrin 8 (ADAM8) is highly expressed in mature granulocytes (27–29), indicating potential relevance in ARDS pathogenesis. ADAM8 shares the typical modular structure of ADAM protease family members, comprising an inhibitory prodomain (Pro); a catalytic metalloproteinase (MP) domain; a disintegrin-like (DI), cysteine-rich, EGF-like domain; a transmembrane region (TM); and a cytoplasmic tail (CD) (30, 31). ADAM8 is activated by autocatalytic removal of the prodomain in the trans-Golgi network and requires homophilic multimerization of at least 2 ADAM8 monomers via their DI domains for in vivo activity (32–34). Active ADAM8 on the cell membrane has been shown to cleave molecules with cell-adhesive and immunological functions, such as VE-cadherin (35), L-selectin (27), PSGL-1 (36), CD23 (37), and CXCL-1 (38), or autoprocess itself to yield a soluble MP module and formation of remnant membrane-associated ADAM8 (30). The intracellular portion of ADAM8 contains Src homology 3 (SH3) domains and binding sites (39), which suggests signaling capability, but little is known about their function.

Here, we investigated the in vivo significance of ADAM8 on neutrophil motility by intravital imaging of the inflamed cremaster muscle and lung and linked the observed neutrophil responses to a noncanonical function of ADAM8 mediated through its intracellular domain. We then investigated the impact of ADAM8 genetic deletion and pharmacological inhibition in mice with *Pseudomonas aeruginosa* infection, which is a common pathogen causing severe pneumonia in hospitalized patients and ARDS (40). Finally, we developed a potential diagnostic approach measuring ADAM8 proteolytic activity in lung fluids of 2 cohorts of patients with ARDS and tested for associations between substrate breakdown, immunological activation, and patient survival.

## Results

### ADAM8 directs neutrophil transmigration in vivo.

**Figure 1:**
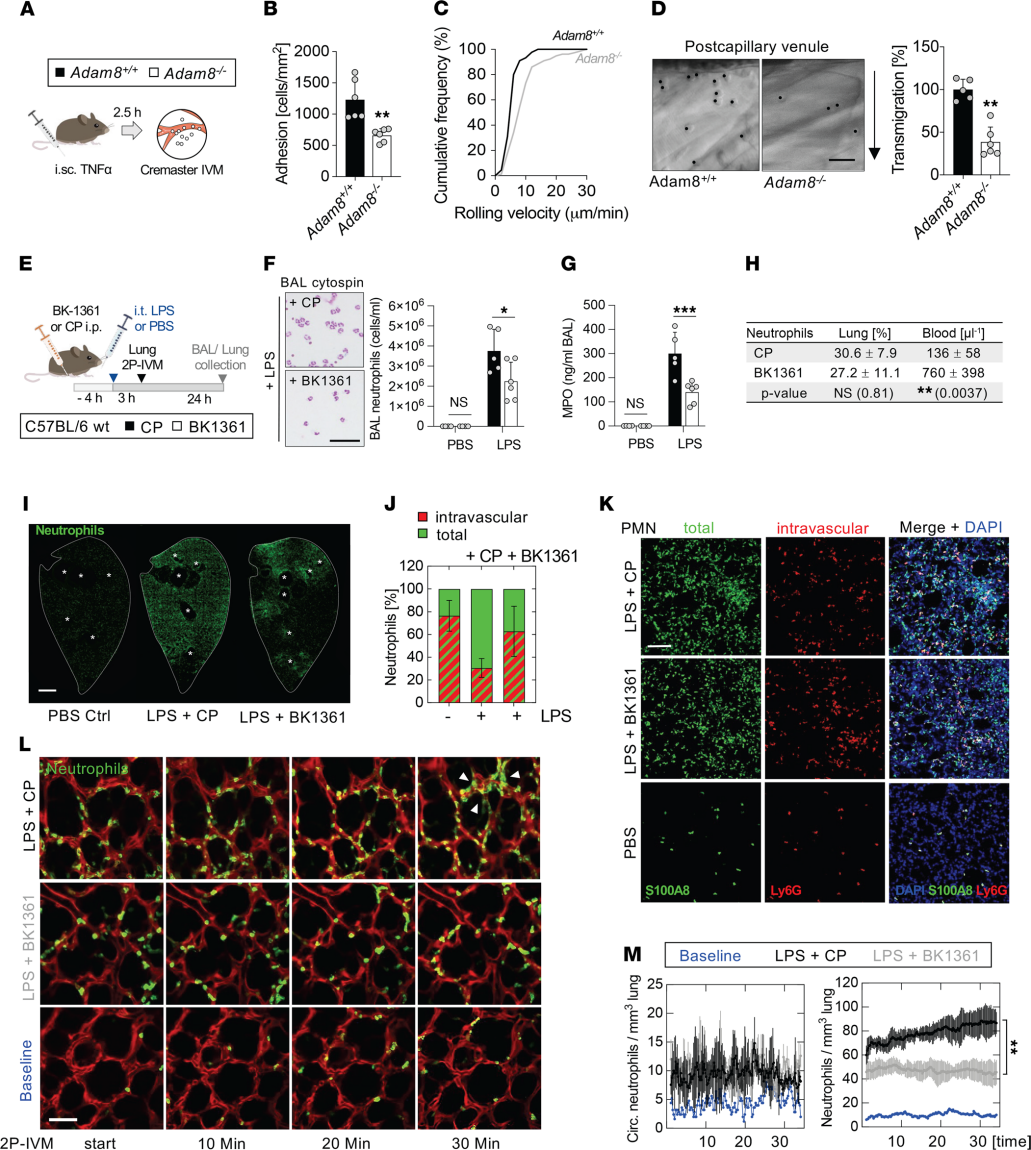
Genetic deletion and pharmacological inhibition of *Adam8* impair neutrophil motility in vivo. (**A**) Schema for cremaster IVM after i.sc. TNF-α. (**B**) Number of cells adherent to endothelium, (**C**) rolling velocities, and (**D**) quantification of transmigrated *Adam8^−/−^* and *Adam8^+/+^* neutrophils (right). Representative fields showing leukocyte extravasation (left); arrow indicates direction of emigration. Scale bar, 25 μm. Data for **B**–**D** are shown as mean ± SD. **, *P* < 0.005; Student’s *t* test. (**E**) Schema for ADAM8 inhibitor experiments after i.t. LPS. (**F**) BAL neutrophils and cytospins (left); scale bar, 25 μm. (**G**) BAL MPO ELISA. (**H**) Quantification of lung tissue and peripheral blood neutrophils. Data for **F**–**H** are mean ± SD. *, *P* < 0.05; **, *P* < 0.01; ***, *P* < 0.001; 2-way ANOVA followed by Holm-Šídák multiple-comparison test. (**I**) Immunostaining of neutrophils (S100A8, green) in 100 μm lung sections. *, large airways. Scale bar, 2 mm. (**J**) Quantification and (**K**) representative images of intravascular (red) and total (green) neutrophils in lung sections at baseline and after i.t. LPS + BK1361 or CP. Ly6G antibody was injected i.v. 10 minutes prior to euthanasia to label intravascular neutrophils; total lung neutrophils were visualized by S100A8 staining (green) and nuclei by DAPI (blue). Neutrophils were quantified from 7 fields/animal (*n* = 5). Data in **J** are mean ± SD, 2-way ANOVA followed by Holm-Šídák multiple comparison test; LPS+CP vs. LPS+BK1361 (*P* = 0.0025); LPS+CP vs. PBS (*P* = 0.008); LPS+BK1361 vs. PBS (NS). Scale bar, 100 μm (**K**). (**L**) Two-photon lung IVM of MRP8-Cre mTmG mice + BK1361 or CP 3 hours after LPS challenge. Representative images of neutrophil influx (MRP8^+^, green). Arrowheads, neutrophil cluster. Scale bar, 50 μm. (**M**) Quantification of neutrophils circulating (track duration < 60 s, left graph) and migrating (track duration > 60 s, right graph) through the lung, *n* = 3. Blue, baseline; gray, LPS+BK1361; black, LPS+CP. **, *P* < 0.01; 2-way ANOVA. i.sc., intrascrotal injection.

ADAM8 is expressed in neutrophils at high levels and is upregulated upon inflammatory stimuli (Supplemental Figure 1, A–G; supplemental material available online with this article; https://doi.org/10.1172/jci.insight.149870DS1) (27, 41). However, little is known about the role of ADAM8 in the neutrophil recruitment cascade in vivo. Consistent with previous results (36, 41, 42), our functional data from isolated mouse *Adam8^–/–^* and *Adam8*^+/+^ bone marrow neutrophils indicated that the absence of ADAM8 impaired neutrophil transmigration (Supplemental Figure 2, A–C) and motility through extracellular matrices (Supplemental Figure 2, E–H) in vitro, while phagocytosis of pathogen particles was not affected (Supplemental Figure 2D). To investigate the contribution of ADAM8 during neutrophil trafficking in vivo, we compared *Adam8*^+/+^ and *Adam8^–/–^* neutrophil responses using intravital microscopy (IVM) in the inflamed cremaster muscle (Figure 1A), a model widely used for studying the stages of the leukocyte adhesion cascade. The adhesion of neutrophils to TNF-α–activated endothelium (Figure 1B) was impaired in *Adam8^–/–^* mice, and the median rolling velocity was increased (*Adam8*^+/+^, 5.7 ± 2.3 μm; *Adam8^–/–^*, 8.8 ± 5.4 μm) (Figure 1C and Supplemental Video 1). Differential interference contrast microscopy images of cremaster venules (Figure 1D, left) and respective quantification (Figure 1D, right) revealed that neutrophil transmigration across the vascular wall was significantly reduced in the absence of ADAM8. Rheological parameters of the analyzed cremaster muscle venules after TNF-α injection did not differ between *Adam8*-deficient mice and littermate controls (Supplemental Figure 3). Together, these data suggest an important role of ADAM8 for neutrophil–endothelial interactions during transendothelial neutrophil migration in vivo.

### Inhibition of ADAM8 multimerization reduces neutrophil influx into the lung.

The conventional paradigm of the neutrophil recruitment cascade is not applicable to all organs, and in the lung, neutrophils sequester in pulmonary capillaries to find a transmigration site instead of rolling as described in systemic postcapillary venules (43, 44). We therefore studied the role of ADAM8 in neutrophil responses in a model of acute lung injury driven by intratracheal (i.t.) lipopolysaccharide (LPS) instillation. To test the impact of blocking ADAM8 on pulmonary neutrophil influx, we treated WT mice intraperitoneally (i.p.) with a small peptidomimetic cyclic inhibitor of ADAM8 multimerization (BK-1361, ref. 33) or the corresponding linear control peptide (CP) with identical amino acid composition prior to inducing acute lung injury by i.t. LPS administration (Figure 1E). We observed a significant decrease of bronchoalveolar lavage (BAL) neutrophils in mice treated with BK-1361 (Figure 1F) compared with control animals, as quantified by differential cell counts and reflected by MPO levels in the BAL (Figure 1G). Leukocyte counts in the blood and lung were not altered by inhibiting ADAM8, yet blood differential values revealed more neutrophils in the blood (Figure 1H). Neutrophil accumulation into the alveolar spaces of lungs was associated with increased ADAM8 levels in the BAL during the acute inflammatory response (Supplemental Figure 4, A–C). In lung sections, we visualized the distribution of neutrophils by immunostaining for S100A8. Animals treated with CP prior to LPS instillation developed global, diffuse alveolar neutrophilic inflammation, while ADAM8 inhibition limited neutrophil recruitment and spared considerable portions of the distal lung parenchyma (Figure 1I). Within the inflammatory lesions, BK-1361 treatment shifted the load of neutrophils from the airspace/interstitial compartments to the intravascular space, as demonstrated by comparing the ratio of intravascular neutrophils (i.v. anti-Ly6G^+^, shown in red) with the number of total neutrophils (S100A8^+^, shown in green) (Figure 1, J and K).

To visualize the effect of ADAM8 blockade on early neutrophil accumulation during LPS-induced lung injury, we used 2-photon lung IVM (45) in MRP8^Cre^ mTmG reporter mice (Figure 1L and Supplemental Video 2). In mice treated with CP, tracking of neutrophils in the lung over time revealed the formation of persistent and stable cell clusters, potentially leading to transmigration events. In contrast, BK-1361 treatment resulted in smaller neutrophil aggregates, which quickly dispersed (Figure 1, L and M, and Supplemental Video 2). The flux of neutrophils circulating through the lung was not significantly altered between the treatment conditions but was higher than in the noninflamed state (Figure 1M). Taken together, we conclude that ADAM8 is important for neutrophil transmigration during pulmonary inflammation in vivo.

### ADAM8 interacts with the motor molecule Myosin1f and modulates neutrophil motility.

**Figure 2:**
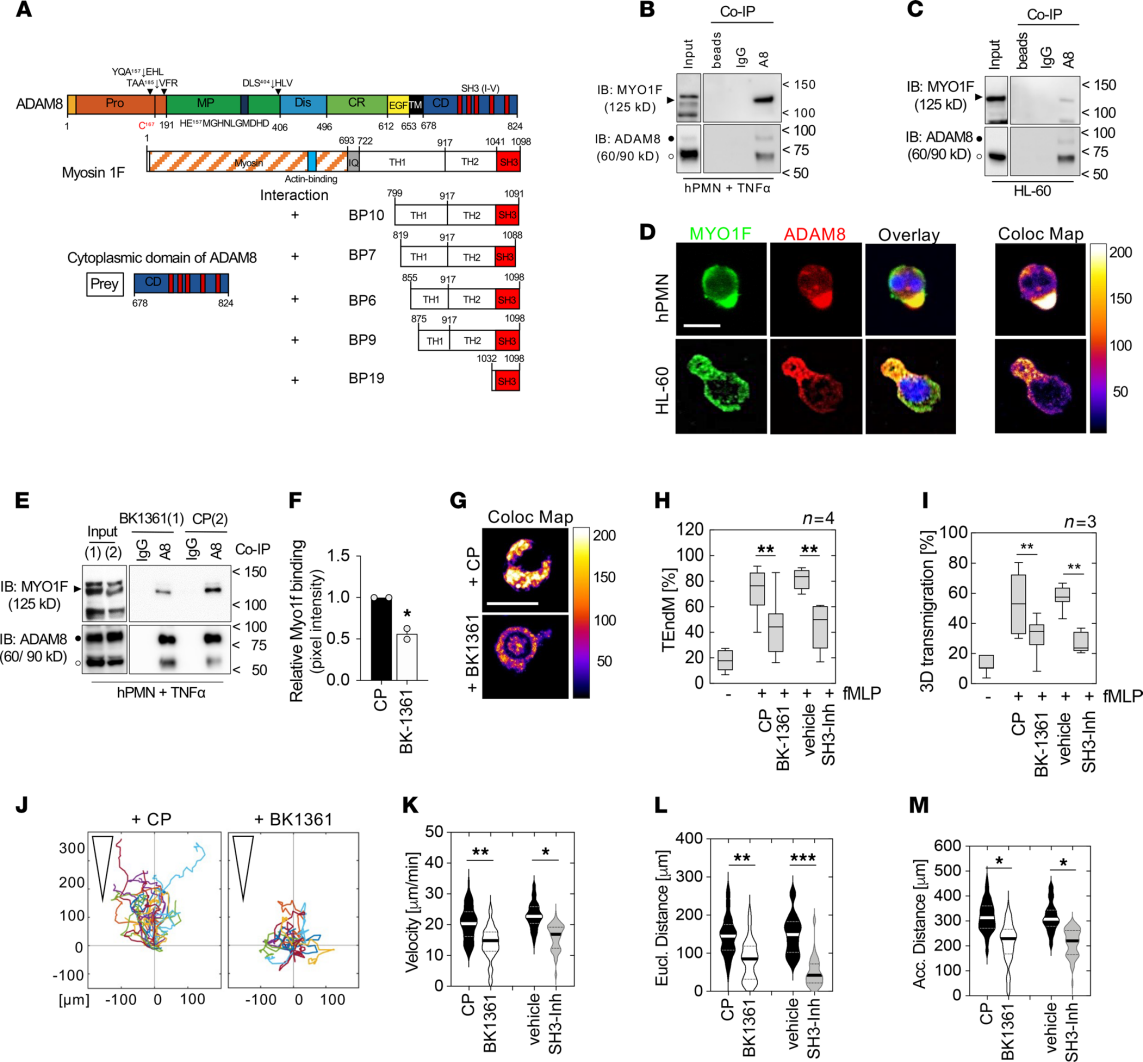
ADAM8 interacts with the actin-based motor protein Myo1f via SH3 domains and modulates cell motility. (**A**) Domain structure of ADAM8 and Myo1f as interaction partner identified by a yeast-2-hybrid screen using a leukocyte library. ADAM8 CD (aa 678–824) was used as prey and the reporter gene indicated interaction (“+”) via the SH3 domain of Myo1f (clone BP19, aa 1032–1098). (**B**) Co-IP of activated human PMN (hPMN) and (**C**) HL-60 cells using polyclonal ADAM8 (A8) antibody coupled to magnetic beads. Negative controls, beads bound to control IgG (IgG); unconjugated beads (beads). (**D**) Immunofluorescence staining against Myo1f (green) and ADAM8 (red) of hPMN and HL-60 cells. Overlay (yellow) and colocalized pixel map (Coloc Map) indicate colocalization in cell protrusions. Scale bar, 10 μm. (**E**) Co-IP of hPMNs + BK-1361 or CP during activation. (**F**) Relative pixel intensity of Myo1f coprecipitated with ADAM8 normalized to input using ImageJ software (NIH). *, *P* < 0.05, Student’s *t* test. (**G**) Colocalization map of hPMNs + BK-1361 or CP as described in **D**. Scale bar, 10 μm. (**H**) Effect of BK-1361 or SH3 domain blockade (SH3-Inh) on fMLP-induced transendothelial migration of hPMNs (*n* = 4 donors). (**I**) Migration across a 3D gel matrix, respectively (*n* = 3 donors). (**H** and **I**) Data are mean ± SD, **, *P* < 0.01; 2-way ANOVA followed by Holm-Šídák multiple-comparison test. (**J**) 3D chemotactic migration of hPMNs preincubated with BK-1361 or CP toward an IL-8 (1 μg/mL) gradient using chemotaxis μ-Slides. Representative trajectory plots. Triangles indicate orientation of the gradient. Violin plots of (**K**) migration velocity and (**L**) mean Euclidian and (**M**) accumulated distance; BK-1361 (white), SH3 inhibitor (gray), control (black). (**K**–**M**) Three independent experiments, 30 cells per experiment were analyzed. *, *P* < 0.05; **, *P* < 0.01; ***, *P* < 0.001; 2-way ANOVA followed by Holm-Šídák multiple-comparison test. fMLP, *N*-formyl-methionyl-leucyl-phenylalanine.

Since ADAM8 regulates inflammatory neutrophil recruitment to the lungs, we sought to determine the mechanism by which ADAM8 could mediate these responses. ADAM8-inhibited neutrophils showed decreased motility in the pulmonary vasculature (Supplemental Video 2), and neutrophil accumulation into the lungs is mainly selectin and integrin independent and is driven by cytoskeletal rearrangement rather than rolling on the endothelium (44). We therefore hypothesized that the interaction of ADAM8 with cell motility-associated molecules via its cytoplasmic domain could be important for injurious transendothelial migration. To identify potential ADAM8 interaction partners in the cytoplasm, a yeast-2-hybrid screen was performed using the intracellular domain of ADAM8 as bait and a Matchmaker cDNA prey fusion protein library from leukocytes. Our screen identified Myosin1f (Myo1f) among others as a potentially novel interacting protein of ADAM8 (Figure 2A). The 5 clones displayed (out of 18 positive hits) showed that the SH3 binding domain of Myo1f (Figure 2A, BP19) was the minimum sequence required for the interaction with ADAM8.

Interestingly, neutrophil extravasation has been shown to depend upon cytoskeletal dynamics regulated by class I myosins (46, 47). To support the ADAM8–Myo1f interaction identified by the yeast-2-hybrid screen, co-immunoprecipitations (co-IPs) using an antibody raised against the ectodomain of ADAM8 were performed. In stimulated human neutrophils (hPMNs) (Figure 2B) and leukocytic HL-60 cells (Figure 2C), Myo1f coprecipitated with ADAM8, and immunofluorescence staining against Myo1f and ADAM8 indicated colocalization in protrusions of neutrophils and HL-60 cells (Figure 2D). Next, we preincubated human neutrophils with BK-1361 prior to stimulation with TNF-α to investigate the effect of blocking ADAM8 multimerization (Supplemental Figure 5) on the ADAM8–Myo1f interaction. BK-1361 reduced the interaction of ADAM8 and Myo1f by about 50% (Figure 2, E–G), whereas treatment with the inhibitory antibody MAB1031 (48) did not affect the interaction (Supplemental Figure 5 and Supplemental Figure 6, A–C). Further, as yeast-2-hybrid screening results suggested an involvement of SH3 domains in ADAM8–Myo1f binding, a 2-aminoquinoline–based SH3 domain inhibitor (49) attenuated the interaction, as we expected (Supplemental Figure 5 and Supplemental Figure 7, A–E). Functionally, treatment of neutrophils with compounds inhibiting ADAM8 or SH3 domain significantly impaired transmigration across the endothelium (Figure 2H and Supplemental Figure 6D) and a thin matrigel layer (Figure 2I and Supplemental Figure 6E), suggesting that both intracellular protein–protein interaction and proteolytic activity contributed to the process of crossing tissue barriers. Tracking of cell movement in 3D matrigel toward an IL-8 gradient indicated that BK-1361 and SH3 domain inhibition decreased cell velocity and migration distance (Figure 2, J–M), whereas MAB1031 did not affect these parameters (Supplemental Figure 6, F–I). Importantly, neutrophil effector functions, including phagocytosis and MPO release, remained unaffected by the inhibition of ADAM8 (Supplemental Figure 8, A–C). These results support our hypothesis that ADAM proteases can interact with SH3 domains of motor molecules and the cytoskeleton to direct the motility of neutrophils.

### ADAM8 inhibition attenuates severe Pseudomonas aeruginosa infection.

**Figure 3:**
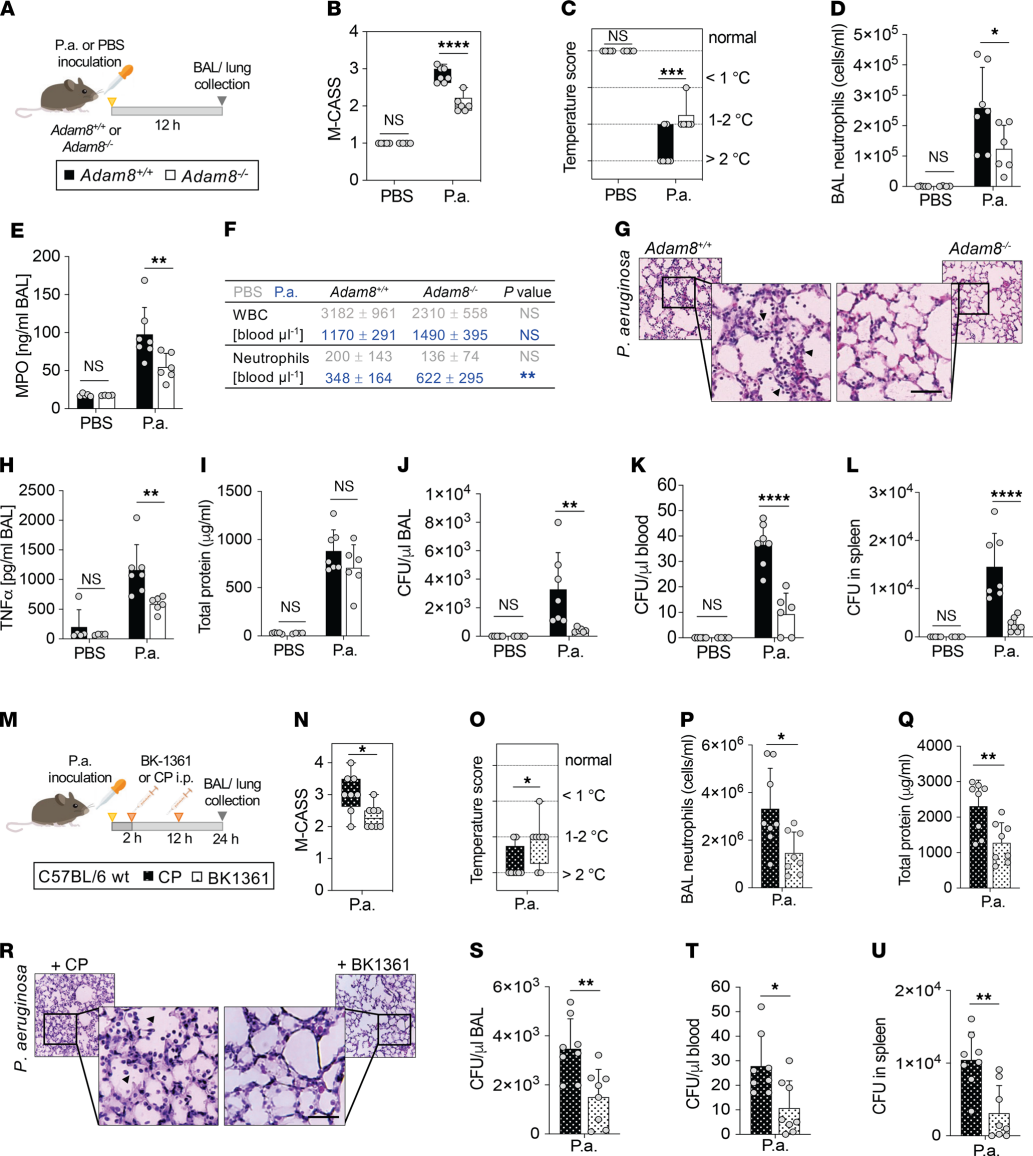
Genetic deletion and pharmacological inhibition of *Adam8* protect mice against severe *P. aeruginosa* infection. (**A**) *Adam8^–/–^* (white bars) or littermate controls (*Adam8^+/+^*, black bars) were intranasally instilled with *P*. *aeruginosa* (strain PA103) or vehicle control (PBS), then sacrificed 12 hours after infection. (**B**) Mouse Clinical Score for Sepsis (M-CASS) and (**C**) temperature scoring were determined in *Adam8^–/–^* (white) and *Adam8^+/+^* (black) mice for *P*. *aeruginosa*–infected and control animals. (**D**) BAL neutrophils and (**E**) MPO levels. (**F**) WBCs and neutrophils in the peripheral blood of *Adam8^+/+^* and *Adam8^–/–^* mice infected with *P*. *aeruginosa* (blue) or instilled with PBS (gray). (**G**) Representative histologic tissue sections of *Adam8^+/+^* and *Adam8^–/–^* mice after 12 hours’ *P*. *aeruginosa* challenge. Arrowheads, neutrophils in the airspaces; scale bar, 50 μm. (See enlarged sections in Supplemental Figure 12.) (**H**) BAL TNF-α and (**I**) total protein. CFU in (**J**) BAL, (**K**) blood, and (**L**) spleen, respectively. (**A** and **B**) Box-and-whisker plots; (**C**–**L**) mean ± SD. *, *P* < 0.05; **, *P* < 0.01; ***, *P* < 0.001; ****, *P* < 0.0001, 2-way ANOVA followed by Holm-Šídák multiple-comparison test. (**M**) C57BL/6J mice were inoculated with *P*. *aeruginosa* and treated with either cyclic ADAM8 inhibitor peptide (BK-1361, dotted white bars) or control peptide (CP, dotted black bars) at 2 and 12 hours after infection, and sacrificed at 24 hours. (**N**) M-CASS and (**O**) temperature scoring. (**P**) BAL neutrophils and (**Q**) total protein. (**R**) Representative histologic tissue sections of mice treated with CP or BK-1361 during *P*. *aeruginosa* challenge. Arrowheads, neutrophils in the airspaces; scale bar, 50 μm. (See enlarged sections in Supplemental Figure 12.) CFU in (**S**) BAL, (**T**) blood, and (**U**) spleen. (**N** and **O**) Box-and-whisker plots; (**Q**–**U**) mean ± SD; individual data points for each animal are plotted as gray dots. * *P* < 0.05; ** *P* < 0.01; Student’s *t* test.

Pulmonary infections with *Pseudomonas aeruginosa* (*P. aeruginosa*) are a common cause of ARDS (40). To evaluate the clinical impact of ADAM8 on acute lung inflammation, we infected the lungs of mice with a genetic deletion of *Adam8* (*Adam8^–/–^*) or lungs of WT littermate controls (*Adam8^+/+^*) for 12 hours with *P. aeruginosa* (Figure 3A), then determined morbidity, lung inflammatory injury, and bacterial load. First, we compared disease severity using the Mouse Clinical Assessment Scoring System for Sepsis (M-CASS, Supplemental Table 1) (50). ADAM8-deficient mice presented with a moderate manifestation of pneumonia, whereas WT control animals developed severe symptoms, as reflected by a higher M-CASS score (Figure 3B). Temperature scoring, as a surrogate marker for survival, indicated hypothermia >2°C in ~70% of the WT animals, while temperature loss ranged between 1°C and 2°C in the majority of *Adam8*-deficient mice (Figure 3C). BAL neutrophil counts were decreased by almost 50% in *Adam8^–/–^* mice versus WT controls (Figure 3, D and E), consistent with our results during LPS-induced lung injury (Figure 1F). *Adam8^–/–^* and WT mice developed leukopenia 12 hours after *P. aeruginosa* infection, but neutrophil counts in the blood of *Adam8^–/–^* mice were increased compared with WT animals, potentially a result of reduced neutrophil transmigration (Figure 3F). Histologically, WT animals developed diffuse alveolar damage with moderate hemorrhage, while in *Adam8^–/–^* mice the inflammatory infiltrates were smaller and without bleeding (Figure 3G). Reduced neutrophilic activation was associated with decreased levels of proinflammatory cytokines, such as TNF-α, in the BAL of *Adam8^–/–^* mice compared with littermate animals (Figure 3H). Alveolar-capillary barrier function (assessed by BAL total protein measurement) was on average improved by 20% in *Adam8^–/–^* mice within 12 hours of *P. aeruginosa* infection (Figure 3I) but was not statistically significant. Interestingly, despite attenuated leukocyte recruitment, we observed less systemic spreading and improved clearance of extracellular *P. aeruginosa*, as reflected by reduced colony forming units (CFU) in the BAL (Figure 3J), the blood (Figure 3K), and the spleen (Figure 3L) of *Adam8^–/–^* mice.

We tested pharmacological ADAM8 blockade in WT mice with pulmonary *P. aeruginosa* infection by treating with BK1361 at 2 hours and 12 hours after inoculation, with euthanasia at 24 hours (Figure 3M). Consistent with our observation in *Adam8^–/–^* mice, the inhibition of ADAM8 reduced the severity of pneumonia (Figure 3, N, O, and R). Accordingly, BAL neutrophils were reduced in mice treated with BK1361 (Figure 3P), while neutrophil counts in the blood were increased compared with animals treated with CP (BK1361, 1156 ± 476; CP, 736 ± 248, *P* value 0.0435). BAL total protein (Figure 3Q) was significantly reduced within 24 hours of *P. aeruginosa* lung infection in the BK1361 treatment group. Importantly, bacterial clearance was not impaired by ADAM8 inhibition, as demonstrated by reduced CFU counts in the BAL (Figure 3S), the blood (Figure 3T), and the spleen (Figure 3U).

### ADAM8 activity significantly contributes to the proteolytic signature of neutrophils.

**Figure 4:**
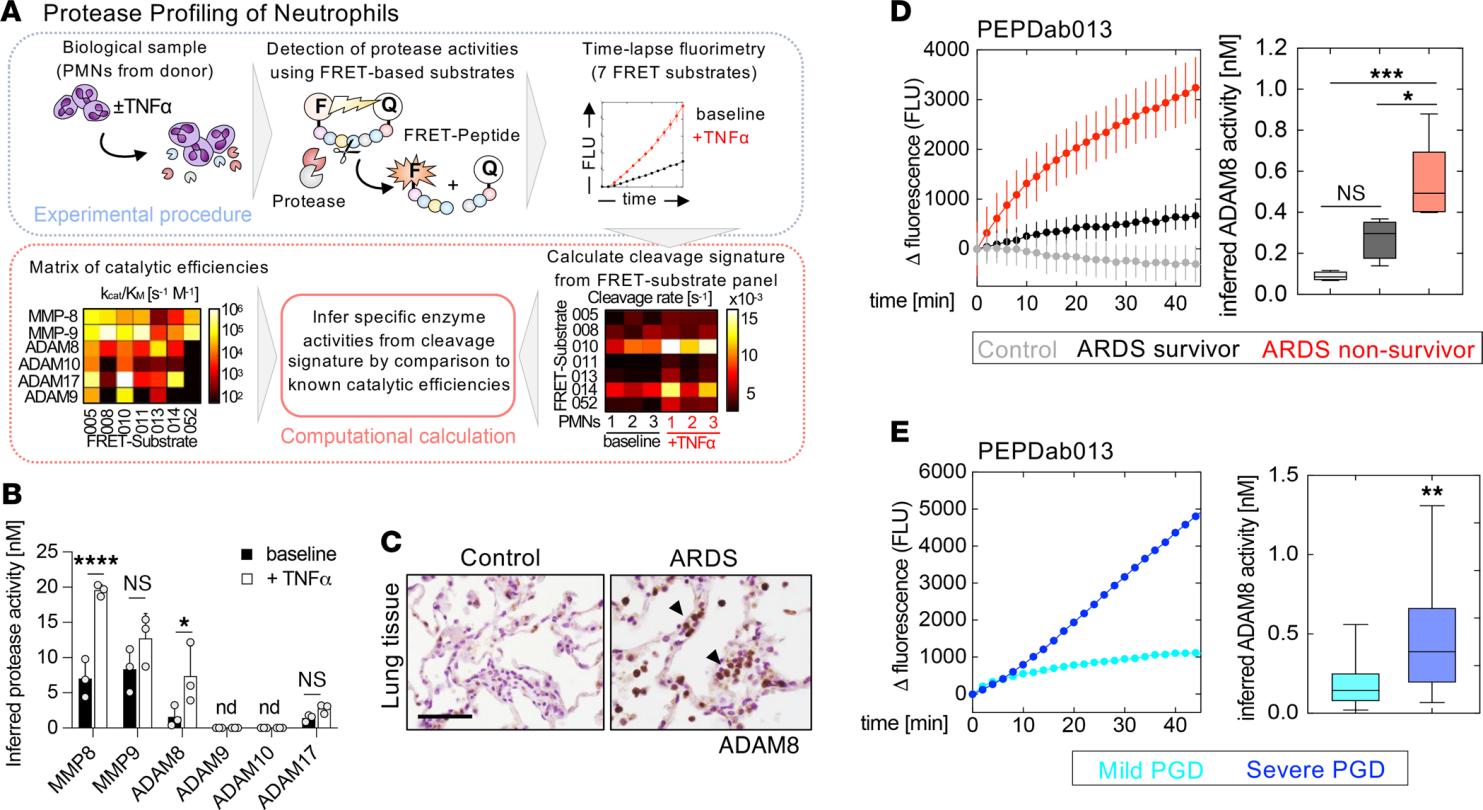
ADAM8 protease is expressed in human leukocytes and detected in lung fluids of patients with ARDS. (**A**) Workflow for protease profiling of PMNs at baseline and during activation with TNF-α (20 ng/mL) using 7 FRET-based peptide substrates (PEPDab 005, 008, 010, 011, 013, 014, and 052). To deduce a profile of specific MMP and ADAM activities, peptide cleavage patterns from 3 independent donors were calculated using a nonlinear kinetic model; proteolytic signatures were compared to a reference matrix of catalytic efficiencies to deconvolute protease identities. (**B**) Proteolytic profiling infers increased activity of known neutrophil MMPs and significant levels of active ADAM8; *, *P* < 0.05; ****, *P* < 0.0001; 2-way ANOVA followed by Holm-Šídák multiple-comparison test. (**C**) Representative images of ADAM8-positive leukocytes (arrowheads, brown color) in lung tissue of patients with ARDS and healthy controls. Scale bar, 50 μm. (See enlarged sections in Supplemental Figure 12.) (**D**) Representative time-lapse fluorimetry of healthy control BAL (gray) and BAL from patients with ARDS from pneumonia (black; ARDS survivor, red; ARDS nonsurvivor) using the most ADAM8-specific FRET reporter, PEPDab013 (mean ± SD of 3 technical replicates); right, box-and-whisker plots of inferred ADAM8 activity in BAL samples of control patients (gray, *n* = 4), ARDS survivors (black, *n* = 5), and ARDS nonsurvivors (red, *n* = 6). *, *P* < 0.05; ***, *P* < 0.001; 1-way ANOVA followed by Tukey’s multiple-comparison test. (**E**) Representative time-lapse fluorimetry of BAL of patients with mild (cyan) or severe PGD (blue) using PEPDab 013 (mean ± SD of 3 technical replicates); right, box-and-whisker plots of inferred ADAM8 activity in BAL samples of mild PGD (cyan, *n* = 16) and severe PGD (blue, *n* = 16). **, *P* < 0.005, Student’s *t* test with Welch’s correction. nd, not detectable.

We analyzed the proteolytic profiles of isolated human neutrophils from healthy donors at baseline and during stimulation with TNF-α to determine how neutrophil catalytic MMP and ADAM activities are modulated during inflammatory activation. To reveal cleavage signatures, we monitored proteolytic activities by time-lapse fluorimetry using a combination of 7 fluorescence resonance energy transfer–based (FRET-based) reporter protease substrates representing known MMP/ADAM cleavage sites (Figure 4A and Supplemental Figure 9, A–F) (51). To increase the specificity of the assay, measurements with 2 protease inhibitors with broad inhibitory spectrum were included into the calculations (Supplemental Figure 9G). Proteolytic profiling of neutrophils deduced the release of active neutrophil collagenase (MMP-8) and gelatinase (MMP-9) (Figure 4B), as reported (22). A significant induction of ADAM8 activity was detected, which was the largest relative increase in activity (~4-fold) among the proteases inferred from the substrate cleavage pattern.

### Cleavage of an ADAM8 reporter substrate in ARDS lung fluid correlates with disease severity.

We confirmed that ADAM8 was primarily expressed by leukocytes in the normal and inflamed human lung by immunostaining of lung tissue sections from either healthy controls or patients with ARDS (Figure 4C). To test for ADAM8 activity in patients with ARDS, we analyzed ADAM8 concentrations in the BAL by ELISA and ADAM8 enzymatic activity as a potential correlate of neutrophil activation in lung fluids. To this end, we selected PEPDab013 as the FRET peptide mostly specific for ADAM8, which is based on the ADAM8 cleavage site in CD23 (Supplemental Figure 9, A and B) (37, 52) and measured breakdown of this substrate by real-time fluorescence increase in BAL samples of patients with and without ARDS from pneumonia. Within 20–30 minutes, activities were robustly detected in the BAL of all patients with ARDS compared with healthy controls (Figure 4D and Supplemental Figure 10, A–C). High substrate cleavage correlated with poor patient survival (Spearman *r* = 0.8504, *P* = 0.0004) (Figure 4D and Supplemental Table 2) in our pilot study. Soluble ADAM8 protein was only detectable by ELISA in BAL samples with high cleavage rates of PEPDab13 (Supplemental Figure 10D), suggesting a better predictive value of the protease assay. However, similar to many endogenous MP substrates, MP FRET substrates are generally cleaved by multiple closely related proteases (51, 52), so breakdown of PEPDab013 may not strictly depend on the presence of ADAM8. By adding broad-spectrum metalloproteinase inhibitors to selected lung fluids, we found that nonmetalloproteinases also contributed to PEPDab013 cleavage. We further tested a reporter substrate that is based on the sequence of pro–TGF-α primarily cleaved by ADAM17 (PEPDab 014, Supplemental Figure 9, A and B), but in contrast to the PEPDab13 substrate with ADAM8 specificity, degradation of the ADAM17-specific substrate by BAL fluid was not associated with disease severity or survival (Spearman *r* = 0.3381, *P* = 0.2844, Supplemental Figure 11).

In addition, we applied the fluorescence cleavage assay using PEPDab013 to a separate ARDS cohort of primary graft dysfunction (PGD) after lung transplantation. In these BAL samples, we found the cleavage rates for PEPDab013 to be significantly higher in patients who clinically presented with severe PGD (corresponding to PGD grade 2 or 3 as defined by the International Society for Heart and Lung Transplantation, ISHLT; ref. 53) compared with patients with no or mild PGD (ISHLT PGD grade 0 or 1) (Figure 4E and Supplemental Table 3), suggesting that increased breakdown of substrate PEPDab013 is associated with PGD severity.

## Discussion

In this study, we provide deep mechanistic insight into the function of ADAM8 in neutrophils during lung infection models and in ARDS. We established a critical role of ADAM8 in inflammatory neutrophil recruitment in vivo by intravital imaging of the inflamed cremaster and lung. Notably, the observed mechanism of action for ADAM8 is not dependent on its enzymatic activity, but rather on nonproteolytic functions of ADAM8 that contribute to these neutrophil responses. Previous in vitro studies were solely based on analyses of leukocytic cell lines and suggested a role for ADAM8 in the leukocyte adhesion cascade, e.g., by PSGL-1 shedding (27) and modulating leukocyte–endothelial interactions through α_L_ integrin regulation (41). However, neutrophil accumulation into the lungs depends on cytoskeletal dynamics and cell stiffening, processes driven by myosin molecular motors rather than by adhesion molecules (44, 46, 47).

**Figure 5:**
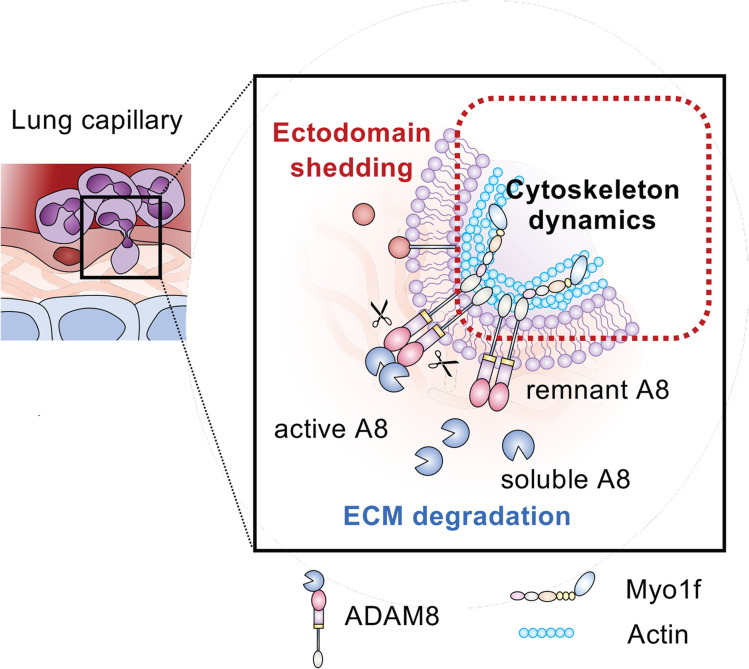
Schematic model of ADAM8–Myo1f interaction during neutrophil transmigration. Neutrophil transmigration requires degradation of ECM and adhesion molecules, as well as cytoskeletal rearrangements. Active ADAM8 has been shown to process membrane proteins with immunological functions (red, ectodomain shedding) and cleave ECM components (blue, ECM degradation) previously (27, 33, 36). Our data suggest a potentially novel contribution of ADAM8 in modulating neutrophil motility by linking its cytoplasmic domain to the cytoskeletal motor protein Myo1f via SH3 domains (red box, cytoskeletal dynamics).

Interestingly, we discovered that ADAM8 interacts with the long-tailed class I myosin Myo1f via its intracellular SH3 domains. Class I myosins are motor proteins linking the actin cytoskeleton to the plasma membrane and serve important functions in the immune system (54). As such, Myo1f is involved in regulating cell adhesion formation (55) and membrane–cytoskeletal crosstalk during phagocytosis (56) and has recently been described as critical for transendothelial migration of neutrophils by deforming the nucleus during diapedesis (46). We found that the inhibition of ADAM8 dimerization using BK-1361 (targeting of the disintegrin domain of ADAM8) or SH3 domain inhibitors attenuated the observed ADAM8–Myo1f interaction and subsequently reduced transmigration and interstitial migration of neutrophils in vitro and in vivo. While the classical dogma is that neutrophils proteolytically “cut” their way through the endothelial barrier to promote extravasation (57), our findings challenge this concept and demonstrate a potentially novel contribution of a metalloproteinase-disintegrin in modulating cell migration by linking its cytoplasmic domain to cytoskeletal motor proteins (Figure 5).

The injurious effect of an exuberant neutrophilic response is well recognized in ARDS development (21). An ideal treatment strategy for ARDS would reduce neutrophil influx without impairing their microbe-clearing and regenerative capacities (18, 20, 21). The promigratory function of ADAM8 in leukocytes has been previously reported in asthma (42, 58) and sterile lung inflammation (41). However, these studies were described in the context of general inflammation and were not focused on neutrophils. Using 2-photon IVM in LPS-induced lung injury, we observed that blocking ADAM8 reduced the formation of stable neutrophil clusters in the lung vasculature, an essential event to allow for neutrophil transmigration. ADAM8 blockade resulted in a reduction of neutrophils in the airspace/interstitial compartment and a retention in the intravascular compartment. It has been shown previously that transmigration triggers neutrophil degranulation (19), which contributes to bystander tissue injury and can result in inflammatory exacerbation. Thus, reducing transmigration and subsequently attenuating the extent of collateral tissue damage may have a favorable effect on lung inflammation.

To validate ADAM8 as a therapeutic target in our study, we chose pulmonary *P. aeruginosa* infection as a well-established preclinical ARDS model. One of the most striking findings when analyzing *Adam8*-knockout mice was that despite a reduced neutrophil recruitment capacity, animal morbidity and bacterial containment were improved. These results prompted us to test the pharmacological inhibition of ADAM8 as a therapeutic strategy for severe *P. aeruginosa*–induced pneumonia in mice. Indeed, we were able to achieve comparable effects by administering BK1361 i.p. after *P. aeruginosa* instillation, which reduced pulmonary neutrophil influx and lung injury and actually improved bacterial containment.

We hypothesize from these data that blocking ADAM8 results in increased numbers of patrolling blood neutrophils that limit systemic bacterial spreading. In the lung parenchyma, alveolar macrophages have been shown to contribute significantly to pathogen clearance during infection, and their antimicrobial functions may compensate for the reduction of neutrophils in the alveoli with ADAM8 inhibition (59). According to our studies, phagocytosis by both macrophages and neutrophils is not impacted by ADAM8 inhibition, which leaves this major defense mechanism against pathogens unaffected. ADAM8 deficiency in leukocytes has further been associated with reduced apoptosis (60), which may explain why despite reduced neutrophil recruitment into the airspaces, extracellular bacteria are more effectively cleared. Ultimately, fewer apoptotic neutrophils require less efferocytosis, which may increase the phagocytic capacities of macrophages for microbes. Inhibition of ADAM8 fine-tunes the immune balance and reduces neutrophil transmigration to an extent that the injurious effect is attenuated, yet bacterial clearance remains efficient.

These data together with previous findings that ADAM8 is upregulated under pathological conditions (31, 61) and is dispensable during homeostasis (62) favor ADAM8 as a promising therapeutic target. Excessive neutrophil recruitment is a common characteristic of patients with fatal lung failure, and attenuating neutrophilic responses by ADAM8 inhibition in combination with standard antibiotic strategies may provide beneficial effects for a wide spectrum of patients with ARDS, but future studies will need to determine if susceptibility to secondary infection increases following the treatment.

We hypothesized that detecting ARDS-associated protease activities under optimized in vitro conditions in lung fluids of patients with ARDS may represent a rapid tool for early recognition and stratification in a critical care setting. We analyzed the proteolytic “fingerprint” of activated human neutrophils by a multiplex protease assay, proteolytic activity matrix assay (PrAMA) (51), and discovered that ADAM8 displayed a strong activation profile among known neutrophil metalloproteinases. Based on our preclinical investigations, we sought to evaluate the clinical utility of measuring ADAM8 levels by ELISA as well as activities by real-time fluorescence increase in BAL samples to monitor immunological activation. Interestingly, breakdown of a moderately ADAM8-specific FRET substrate correlated with disease severity in BALs of patients with 2 etiologies of ARDS (pneumonia and PGD after lung transplantation). Importantly, tracking proteolytic activities is rapid (20–30 minutes) and has an experimental workflow simpler than ELISA (63). Although MP FRET substrates, similar to endogenous MP substrates, are generally cleaved by multiple closely related proteases (51), and thus not all enzymatic activity detected is attributable to ADAM8, tracking of proteolytic activities in lung fluids using a fluorescent substrate that covers ADAM8 activity could be a useful tool to rapidly monitor and stratify ARDS patients. We propose that by using a quick test system and fast responding markers, such as proteases, the acute onset and dynamic nature of ARDS may be captured more precisely. This could help identify patients with high-risk profiles early in the clinical course, which would allow for closer monitoring and earlier supportive intervention.

The limitations of monitoring ADAM8 activity in ARDS should be considered. Our cohorts provide only proof-of-concept data, there is an uncertain activity loss due to storage and thawing of samples, and there is currently a lack of highly specific MP-FRET substrates. To address these issues, more clinical studies are required to evaluate proteolytic signatures for ARDS phenotyping against existing approaches for ARDS endotyping. Nevertheless, advancing the tracking of protease activities to point-of-care tests could be a powerful tool for quick risk profile assessment and guidance for therapeutic interventions. In conclusion, we provide evidence that ADAM8 signaling drives neutrophil recruitment in ARDS and constitutes a specific target for ARDS therapeutic development.

## Methods

### Animals.

Mice were housed and bred under specific pathogen–free conditions, and animal experiments were approved by the local authorities, conforming with ethical principles and animal protection laws at the respective location (reference 2.4.2.2 — 42/2018, State Agency Environment and Consumer Protection Saarland, Germany; IACUC protocol AN186887-01A, University of California, San Francisco, San Francisco, California, USA). *Adam8^−/−^* and *Adam8^+/+^* mice were maintained on a C57BL/6J background for at least 10 generations, and C57BL/6J WT mice for ADAM8 inhibitor studies were purchased from Jackson Laboratory. To track neutrophils in the lung, MRP8^Cre^ (stock 021614, Jackson Laboratory) mice were crossed with ROSA^mT/mG^ (stock 007676, Jackson Laboratory) reporter mice. Weight-matched male and female mice aged 6–10 weeks were randomly allocated to experimental groups within a litter.

### IVM of the mouse cremaster muscle.

Mouse cremaster muscle preparation for intravital imaging was performed as described previously (64). Briefly, 500 ng of TNF-α (R&D Systems) was injected intrascrotally in male *Adam8^+/+^* or *Adam8^–/–^* mice 2 hours before visualization of the microcirculation using a transillumination intravital microscope (Axioskop). Rolling and attachment of leukocytes were recorded by a digital camera (ORCA Flash 4.0, Hamamatsu) for analysis in ImageJ. Leukocyte flux was quantified by counting the number of cells that rolled past a fixed perpendicular line over 1 minute (cells/min); rolling velocity was calculated from the distance a leukocyte travels in 2 seconds (μm/s). Adherent cells were classified as those being stationary for 30 seconds. Leukocyte extravasation was visualized by near-infrared reflected-light oblique transillumination microscopy (Axioskop); transmigrated cells were quantified in 5 fields within an area of 150 × 100 μm to each side of a postcapillary vessel. For WBC count, blood was collected from the left ventricle, and leukocytes were counted after red blood cell lysis using an automated cell counter (Countess 3, Invitrogen).

### Lung 2-photon IVM.

To observe neutrophil trafficking in the lung, we performed 2-photon IVM as described previously (45, 65). Mice were anesthetized with a mixture of ketamine (100 μg/g BW) and xylazine (8–12 μg/g BW) and secured on a feedback-controlled, heated microscope stage. A tracheostomy was performed; mice were intubated with PE-90 tubing and connected to a MiniVent mouse ventilator (Harvard Apparatus) using a tidal volume of 10 μL/g BW of air (21% O_2_) at a respiratory rate of 125 breaths/min and a positive-end expiratory pressure of 3 cmH_2_O. For anesthesia maintenance, isoflurane was continuously administered, and anesthesia depth was monitored by assessing canthal reflex and the presence of spontaneous movement. To compensate for fluid loss during the procedure, 250 μL of 0.9% saline solution was injected i.p. every hour. To insert the thoracic window, mice were placed in the right lateral decubitus position, and a surgical incision into the skin across the left side was made. Muscle layers were bluntly dissected aside to expose the rib cage. The intercostal space between ribs 4 and 5 was carefully opened through the parietal pleura using straight ophthalmic scissors to expose the surface of the left lung lobe. A flanged thoracic window with an 8 mm coverslip was inserted between the ribs and secured to the stage using a set of 2 optical posts and a 90° angle post clamp (Thor Labs) (65). Suction of 20–25 mmHg (Amvex Corporation) was applied to gently immobilize the lung. The 2-photon microscope objective (25×/1.1 NA Plan Apo LWD water immersion, Nikon) was positioned over the thoracic window, and intravital imaging was performed for 2 hours using a Nikon A1R upright laser scanning confocal microscope (UCSF Biological Imaging Development Core). Mice were euthanized at the end of the procedure. Video sequences were analyzed using Imaris 9.6 software (Oxford Instruments).

### LPS-induced lung injury model.

Mice were anesthetized using ketamine (50–80 μg/g BW) and xylazine (8–12 μg/g BW) i.p. and i.t. instilled with LPS (3.75 μg/g BW) from *E*. *coli* (O111:B4, MilliporeSigma) dissolved in PBS or with sterile PBS for control. The ADAM8 inhibitor BK-1361 (cyclic conformation, sequence RLsKDK, “s” as d-serine; ref. 33) or a CP (linear peptide of identical amino acid composition) was administered i.p. (10 μg/g BW) 4 hours before i.t. LPS instillation. At the endpoint of LPS challenge, mice were euthanized, and a BAL sample was obtained by cannulation of the trachea. Blood was collected from the vena cava inferior for differential cell counting. Lungs were perfused by injecting 10 mL of ice-cold PBS via the right ventricle, the right bronchus was tied with a suture, and the lung was snap-frozen and cryoground for gene and protein expression analysis. The left lung was slowly filled with fixative (20 mmHg inflating pressure), sutured to prevent backflow, and transferred in 10% formalin solution for histology. The number of neutrophils in the BAL was quantified by differential cell counting of BAL cytospin preparations, flow cytometry, and detection of MPO in the BAL using ELISA (R&D Systems, DY3667). Soluble ADAM8 levels (Biomatik, EKU02052) and the release of cytokines (CXCL-1, R&D, DY453; TNFα, R&D Systems, DY410) into the BAL were quantified by ELISA kits. Total BAL protein concentration was determined by Pierce BCA Protein Assay (Thermo Fisher Scientific).

### P. aeruginosa infectious lung injury model.

Mice were anesthetized as described above and inoculated with 10^6^ CFU *P. aeruginosa* (strain PA103, original source not available) dissolved in PBS or with sterile PBS for control. The experiment was terminated 12 hours after *P. aeruginosa* challenge in the ADAM8-deficient mice. Animals subjected to ADAM8 inhibitor or control treatment were injected i.p. (10 μg/g BW) at 2 and 12 hours after *P. aeruginosa* infection with euthanasia at 24 hours. To assess animal morbidity during the infection, mice were closely monitored according to the Gesellschaft für Versuchstierkunde/Society of Laboratory Animal Science welfare scoring and M-CASS (50), including parameters such as weight, temperature, heartbeat and breath frequency, general condition, and behavior (Supplemental Table 1). Mice were euthanized at designated endpoints, and blood, BAL, and lungs were collected and analyzed as described above. To evaluate bacterial load and spreading after pulmonary *P. aeruginosa* infection, CFU were determined in blood, spleen, and BAL fluid by serial dilution and subsequent growing on tryptic soya agar plates prepared in-house.

### Isolation of neutrophils from mouse bone marrow and human peripheral blood.

Neutrophils were isolated from bone marrow of *Adam^+/+^* and *Adam8^–/–^* mice using a discontinuous Percoll gradient (52%/64%/72%) as described previously (66). For human neutrophil isolation, peripheral blood was collected from healthy donors and anticoagulated with acid citrate dextrose. Neutrophils were purified by dextran sedimentation followed by low-density Ficoll-Histopaque (1.077 g/mL, MilliporeSigma) gradient centrifugation of the leukocyte-rich plasma. Cells were kept in DMEM supplemented with 10% FCS, 25 mM HEPES, 2 mM glutamine, and 1% antibiotics, until they were used in functional assays the same day.

### Yeast-2-hybrid screening.

The cDNA fragment encoding the intracellular domain of human ADAM8 (aa 1–824) corresponding to amino acids 675–824 was subcloned into the yeast expression plasmid pACT2 in-frame to the GAL4 DNA binding domain. Correct reading frames were confirmed by sequencing. For screening of interaction partners, the Matchmaker human leukocyte cDNA library (CLONTECH) was used. Preparation of all media and reagents and all manipulations of the yeast strains HF7c and SFY as well as the β-galactosidase filter assay were performed according to the Matchmaker GAL4 Two-Hybrid System 3 protocol (CLONTECH).

### Synthesis of SH3 domain inhibitors.

SH3 domain inhibitors were synthesized as described previously (49). Cpd_A was compound 12a in that previous report, and cpd_B was compound 12c.

### In vitro 3D chemotaxis assay.

Analysis of 3D chemotaxis was performed in Ibidi Chemotaxis μ-Slides according to the manufacturer’s protocol. Briefly, *Adam8*^–/–^ and *Adam8*^+/+^ neutrophils isolated from the bone marrow were seeded into matrigel (Corning, 200 μg/mL) at a density of 5 × 10^6^ cells/mL. Patent colored CXCL-1 (1 μg/mL) was applied in 1 reservoir of the chemotaxis slide to establish a chemoattractant gradient. Time-lapse video microscopy was performed every 30 seconds at 37°C and 5% CO_2_ for 30 minutes using a BioTek live cell imager. Accordingly, human neutrophils were isolated from peripheral blood and preincubated for 2 hours with ADAM8 inhibitors and respective controls (BK-1361 and CP; 1 μg/mL, MAB1031 [R&D Systems] and IgG control; 10 μg/mL) before seeding into matrigel and exposure to an IL-8 gradient (1 μg/mL) on Ibidi Chemotaxis μ-Slides as described above.

### In vitro 3D transmigration and transendothelial migration assay.

Transmigration was analyzed using Transwell cell culture inserts (Corning, pore size 8 μm) coated with either a thin layer of matrigel (Corning, 200 μg/mL) for 3D transmigration or a monolayer of endothelial cells (brain-derived b.End3 endothelial cells, ATCC CRL-2299; human microvascular endothelial cells, HMEC-1, ATCC CRL-3243) for transendothelial migration. Endothelial cells were activated with 20 ng/mL TNF-α for 2 hours prior to transmigration assays. A total of 2 × 10^5^ murine neutrophils or human peripheral neutrophils were seeded in the upper compartment of the Transwell, and transmigration was stimulated by adding CXCL-1 (100 ng/mL) or fMLP (100 nM) to the lower compartment. Neutrophils were allowed to transmigrate for 45 minutes at 37°C and 5% CO_2_, and transmigrated cells in suspension were counted using an automated cell counter.

### Phagocytosis assay.

Primary human neutrophils or monocytes were infected with 100 MOI pHrodo *E*. *coli* particles (Thermo Fisher Scientific) and incubated for 2 hours at 37°C and 5% CO_2_. Subsequently, phagocytic uptake was measured by recording fluorescence intensities using a Sony SH-800 flow cytometer. Quenching to exclude particles adhering on the cell surface is not required given that pHrodo only fluoresces after acidification, e.g., in the lysosomal compartment.

### Flow cytometry.

To analyze the surface expression of ADAM8 on human neutrophils and PBMCs, cells were stained with a mouse monoclonal antibody against the ectodomain of ADAM8 (2.5 μg/1 million cells, R&D Systems) or respective IgG2b (R&D Systems) isotype control followed by incubation with an anti-mouse Alexa Fluor 647–conjugated antibody (5 μg/mL) as described previously (41). To determine the cell populations in the murine BAL or the peripheral blood, samples were stained using the following antibodies: eFluor450-conjugated CD11c, FITC-conjugated CD11b, PE-conjugated CD86, APC-conjugated Ly6G, PerCP-Cy5.5–conjugated CD45, and PE-Cy7–conjugated F4/80 (Thermo Fisher Scientific, BioLegend). (See complete antibody information in Supplemental Table 4.) Fluorescence intensities were recorded using Sony SH-800 flow cytometer, and data were analyzed off-line using FlowJo 10.5.3 software (Tree Star, Inc.).

### Immunofluorescence and confocal microscopy.

Analysis of ADAM8–Myo1f colocalization was performed using confocal microscopy. Human neutrophils were allowed to adhere to poly-d-lysine–coated coverslips and stimulated with 20 ng/mL TNF-α for 30 minutes at 37°C. HL-60 cells (ATCC CCL-240) were differentiated with 1.5% DMSO for 5 days before seeding on coverslips. Cells on coverslips were fixated with 4% paraformaldehyde for 20 minutes, permeabilized with 0.25% Triton X-100, and blocked with 5% BSA. For double immunofluorescence, a sequential staining protocol was used: ADAM8 was labeled using a goat polyclonal antibody against the ectodomain (1:100; R&D Systems, AF1031); for detection of Myo1f, a rabbit polyclonal antibody directed against the N-terminal domain (1:100; Biorbyt, orb221550) was used. For fluorescence staining, Alexa Fluor donkey anti-goat (AF568) and mouse anti-rabbit (AF488) secondary antibodies (1:500; Abcam) were applied, and Hoechst 33342 was applied for nuclear counterstaining. Images were acquired with a Leica SP8X upright confocal microscope and a Leica HC PL APO 40×/1.30 oil objective using 3 lasers at excitation wavelengths 350 nm, 488 nm, and 561 nm.

### Immunohistochemistry.

Immunostaining was performed on formalin-fixed, paraffin-embedded murine or human tissue. In brief, the paraffin blocks were sliced into 5 μm thick sections, deparaffinized with xylene, and rehydrated with decreasing concentrations of ethanol. For heat-induced epitope retrieval, tissue slides were incubated in Antigen Unmasking Solution (Vector Laboratories) in a steamer and cooled to room temperature for 60 minutes. Endogenous peroxidases were quenched with 0.3% hydrogen peroxide (Thermo Fisher Scientific) for 30 minutes. Avidin/Biotin Blocking Kit (Vector Laboratories) was used to block endogenous biotin receptors and avidin binding sites. Primary antibodies were applied overnight at 4°C in a humidified chamber (1:100, ADAM8 [R&D Systems, AF1031]; 1:100, mADAM8 [Biorbyt, orb4376], 1:100 MPO [R&D Systems, AF3667]), followed by incubation with the respective secondary antibody (1:200; Vector Laboratories) for 45 minutes at room temperature. For detection of biotinylated antibodies, the slides were incubated with Vectastain ABC reagent and developed under the microscope by applying diaminobenzidine tetrahydrochloride solution (Vector Laboratories). Finally, the sections were counterstained with hematoxylin (Vector Laboratories), dehydrated in increasing ethanol concentrations and xylene, and coated with xylene-based mounting medium (Thermo Fisher Scientific) and a coverslip.

### Thick floating lung section immunofluorescence.

Following LPS challenge, mice were i.v. injected with 5 μg Ly6G antibody (1A8, host: rat, BioXCell) 10 minutes prior to euthanasia to exclusively label intravascular neutrophils. The tissue collection and staining procedure was adapted from previous reports (67, 68). Coarse scissors were used to carefully open the chest without cutting into the lung. The right bronchus was tied off with a suture, and the lung was excised below the ligature and used for biochemical applications. A perfusion catheter was inserted into the trachea, and the left lung was inflated with 1% paraformaldehyde at a pressure of 20 mmHg. To maintain the extension of the lung, the left bronchus was tied, and the lung was removed (cut above the ligature) and transferred into fixative for 1–2 hours at room temperature. Following mild fixation, the lung was cut transversely into 2 portions with a scalpel, such that the main bronchus, pulmonary artery, and pulmonary vein could be visualized in cross section. The lung portions were washed in PBS and cryoprotected by soaking with 30% sucrose in PBS at 4°C overnight. The next day, lung halves were embedded facedown in OCT medium in disposable plastic beakers (Thermo Fisher Scientific 02–544-30) and frozen as cryostat blocks on dry ice. Lung sections of 100 μm thickness using the block-trimming function of a Leica cryostat cooled to −22°C were cut. Each thick section was quickly transferred into PBS in a 6-well dish, until the OCT dissolved and the lung sections floated. Lung sections were carefully washed in PBS + 0.3% Triton X-100, then incubated with primary antibody (1:500, S100A8, host: goat, AF3059, R&D Systems) in blocking buffer (PBS + 0.3% Triton X-100 + 0.3% BSA + 10% donkey serum) overnight at room temperature. After washing the next day, the floating sections were stained with secondary antibodies (1:500, anti-goat AF488 [Jackson ImmunoResearch, 705-545-003]; 1:500, anti-rat Cy3 [Jackson ImmunoResearch, 712-165-153]) in PBS and 0.3% Triton X-100 overnight. The wash step was repeated the next day, and the tissue was again briefly fixed with 1% paraformaldehyde for 5 minutes. The fixative was washed off and the section were mounted on glass slides using Vectashield mounting medium with DAPI. Images were acquired with a Nikon A1R upright laser scanning confocal microscope with a 25×/1.1 NA Plan Apo LWD water immersion objective using 3 lasers at excitation wavelengths 350 nm, 488 nm, and 561 nm.

### Human ARDS biosamples.

BAL fluids were obtained from healthy controls or patients with ARDS with informed written consent of the participants and ethics committee (Az 58/15 and Az. 87/12)/IRB (No. 13-10738) approval at the Universities of Giessen and Marburg Lung Center (Department of Internal Medicine II, Giessen/Department of Pulmonary and Critical Care Medicine, Marburg) and the Department of Medicine, Division of Pulmonary, Critical Care, Allergy and Sleep Medicine at the University of California, San Francisco (UCSF). BAL was collected early in the disease course (within 48 hours of ARDS onset for the pneumonia cohort; on the first postoperative day in the PGD cohort), kept at 4°C after sampling, and processed in less than 2 hours. Samples were separated into cellular components and cell-free supernatants by centrifugation for 10 minutes at 350*g*, then stored at –80°C. Lung tissue collection from brain-dead organ donors excluded for transplantation who were diagnosed with ARDS or had no ARDS (control) was IRB approved (HSC-MS-08-0354) by the University of Texas Health Science Center at Houston. A central piece (~1 × 1 cm) was obtained from the explanted lung lobes and fixed with 10% formalin on site within 20 minutes as described previously (69).

### Profiling of protease activities using PrAMA.

Commercially available fluorescence-based polypeptides of roughly 7–12 amino acids that are based on endogenous metalloproteinase substrate cleavage sites were used to determine multiple activities in biological samples. These polypeptides are flanked by FRET paired fluorophores, which exhibit quenched fluorescence until proteolytic cleavage of the peptide substrate occurs (52). Thus, protease activity dynamics can be observed by the change of fluorescence over time. Human neutrophils and biological specimens from patients with ARDS were tested for MMP and ADAM activity by PrAMA technique using FRET-based polypeptide substrates PEPDab005 [Dabcyl-LAQAPhe(homo)RSK(5FAM)-NH_2_], PEPDab008 [Dabcyl-PChaGC(Me)HAK (5FAM)-NH_2_], PEPDab010 [Dabcyl-SPLAQAVRSSK(5FAM)-NH_2_], PEPDab011 [Dabcyl-GPLGMRGK (5FAM)-NH_2_], PEPDab013 [Dabcyl-HGDQMAQKSK(5FAM)-NH_2_], PEPDab014 [Dabcyl-EHADLLA VVAK(5FAM)-NH_2_], and PEPDab052 [Dabcyl-APFEMSAK(FAM)-NH_2_] obtained from BioZyme Inc (51, 70) with different specifity toward metalloproteinase members. PrAMA analysis was performed as described previously (51, 70). Briefly, a final substrate concentration of 10 μM in 50 μL of activity buffer (1 μM ZnCl_2_, 20 mM Tris-HCl pH 8.0, 10 mM CaCl_2_, 150 mM NaCl, 6 × 10^−4^% Brij-35) was incubated with 5 × 10^5^ unstimulated or stimulated (TNF-α, 20 ng/mL) human neutrophils in 50 μL serum-free, phenol red–free medium for time-lapse fluorimetry on a 96-well white opaque plate. Each sample was run in duplicate. To confirm the observed activities were attributable to MMPs and ADAMs, the broad-spectrum metalloproteinase inhibitor batimastat (MilliporeSigma) and cOmplete Protease Inhibitor (Roche) were included in the analysis. Fluorescence units were monitored every 2 minutes for 4 hours at 37°C with a BioTek microplate reader using excitation and emission wavelengths of 485 and 530 nm. A nonlinear model was used for curve fitting as described earlier (51), the signal of a negative control (FRET substrate only) was subtracted, and time-lapse fluorimetry data were normalized to a positive control (0.01% trypsin). Specific protease activities were inferred by comparing the substrate cleavage rates for each biological sample to a matrix of known substrate specificities for ADAM8, ADAM9, ADAM10, ADAM17, MMP-8, and MMP-9. All calculations and statistical evaluation of data were conducted using Matlab (2018a, MathWorks).

### Data and materials availability.

All relevant data associated with this study are included in the paper or the supplemental materials. The GTEx human biospecimen reference database (GTEx Analysis Release V8, ENSG00000151651.15) is publicly available.

### Statistics.

All data were analyzed and plotted using GraphPad Prism 7 software (GraphPad Software Inc) and are shown as means ± SD or means ± SEM as indicated. Results plotted as box-and-whisker plots represent median, first and third quartiles, and upper and lower maxima. Sample size was not predetermined; independent biological replicates (*n* values) are specifically defined in the figures and/or figure captions. Statistical significance for pairwise comparison of experimental groups was determined using unpaired 2-tailed Student’s or Welch’s *t* test as indicated. For multiple comparisons for 3 or more independent groups against each other, 1-way ANOVA was performed, and data were adjusted for the multiple testing using Tukey’s multiple-comparison test. Two-way ANOVA with Holm-Šídák multiple-comparison test was used to compare independent variables between 2 experimental groups. *P* values less than 0.05 were considered statistically significant.

### Study approval.

All animal experiments were approved by the IACUC at UCSF or the State Agency Environment and Consumer Protection Saarland, Germany. All human subjects were enrolled after receipt of informed written consent in a protocol approved by the UCSF Committee for Human Research (IRB) or the ethics committee at the Universities of Giessen and Marburg Lung Center.

## Author contributions

CC and JWB conceived the original idea. CC, JWB, and MRL supervised the project. CC, SJC, MRL, AM, DY, and JWB planned the experiments. CC, SJC, AM, DY, LC, and US carried out and analyzed the experiments. BP performed yeast-2-hybrid analysis. JL, NKB, AA, KMW, JJT, MM, CV, and LQ contributed to sample preparation. SFMH helped with computational analysis. JLB, AZ, HKE, MRL, and JWB provided research equipment. HKQ, WB, SH, NS, BS, JPS, and MRL provided patient specimens. SCM and ZTB synthesized and provided SH3 domain inhibitors. CC, SJC, MRL, AM, AZ, DY, and JWB contributed to the interpretation of the results. CC wrote the manuscript; DY, JLB, AM, AZ, SJC, MM, CV, MRL, and JWB provided critical feedback; and CC, MRL, and JWB revised the manuscript. All authors agreed to the final version of the manuscript.

## Supplementary Material

Supplemental data

Supplemental video 1

Supplemental video 2

## Version 1 02/08/2022

Electronic publication
