# Supplementary material for: ADAM8 signaling drives neutrophil migration and ARDS severity
Source: JCI Insight. 2022 Feb 8;7(3):e149870. doi: 10.1172/jci.insight.149870 (PMC8855804; doi:10.1172/jci.insight.149870)
Supplement: Supplemental data [file jciinsight-7-149870-s033.pdf]

Supplementary Materials

Listing of the CAPSys Study group.

Fig. S1. ADAM8 is primarily expressed and released by neutrophils during acute inflammation.

Fig. S2. Functional analysis of Adam8+/+ and Adam8-/- neutrophils.

Fig. S3. Rheologic parameters of cremaster venules analyzed in Figure 1A-D.

Fig. S4. ADAM8 levels during LPS-induced lung inflammation are dependent on neutrophils.

Fig. S5. Effect of ADAM8 ectodomain inhibition using a monoclonal antibody.

Fig. S6. SH3 domain inhibition attenuates the ADAM8-Myo1f interaction.

Fig. S7. Schematic model of drug actions on ADAM8 and Myo1f.

Fig. S8. Effect of ADAM8 inhibition on MPO release and phagocytosis in neutrophils.

Fig. S9. Proteolytic profiling of human neutrophils.

Fig. S10. PEPDab013 cleavage and ADAM8 protein levels in lung fluids of ARDS patients and healthy controls.

Fig. S11: PEPDab014 cleavage in lung fluids of ARDS patients and healthy controls.

Fig. S12. Lower power field magnifications of histologic sections.

Table S1. Surrogate endpoint scoring system for assessing disease severity in mice.

Table S2. Summary of clinical data of patients with ARDS due to pneumonia.

Table S3. Summary of clinical data of patients with ARDS due to Primary Graft Dysfunction (PGD).

Table S4. Table of Key Resources.

Video S1. Representative intravital microscopy sequences of the mouse cremaster muscle of $ADAM8^{+/+}$ and $ADAM8^{-/-}$ mice 2.5h after TNFα injection. Scale bar, 25μm.

Video S2. Representative 2-photon lung intravital microscopy of MRP8-Cre x mTmG mice. Neutrophils (MRP8+, green) were tracked over 30 Minutes in the lung (red) at baseline, or in mice treated with either the ADAM8 inhibitor BK-1361 or a control peptide during acute LPS-induced lung inflammation. Scale bar, 50μm.

Listing of the CAPSys Study group

The following individuals and institutions constitute the CAPSys Study Group:

Petra Creutz, Aline leClaire, Agata Mikolajewska, Markus Vogtmann, Michaela Niebank, the ICU-Team, Carmen Garcia, Norbert Suttorp, Martin Witzenrath, Charité Universitätsmedizin Berlin, Medizinische Klinik m. S. Infektiologie und Pneumologie, Charitéplatz 1, 10117 Berlin; Sven Gläser, Henning Kahnert, Vivantes Klinikum Spandau, Kard., Pneum. und kons. Intensivmedizin, Neue Bergstraße 6, 13585 Berlin; Felix Rosenow, Universitätsklinikum Münster, Kardiologie 1, Albert-Schweitzer-Campus 1, 48149 Münster; Markus Scholz, Universität Leipzig, Institut für Medizinische Informatik, Statistik und Epidemiologie, Haertelstraße 16-18, 04107 Leipzig; Julio Vera-González, Universitätsklinikum Erlangen, Hautklinik, Hartmannstraße 14, 91052 Erlangen; Bernd Thomas Schmeck, Universitätsklinikum Gießen und Marburg, Standort Marburg, Klinik für Pneumologie, Hans-Meerwein-Straße 2, 35043 Marburg; Markus Löffler, Universität Leipzig, Institut für Medizinische Informatik, Statistik und Epidemiologie, Haertelstraße 16-18, 04107 Leipzig; Uwe Völker, Sven Hammerschmidt, Ernst-Moritz-Arndt-Universität Greifswald, Interfakultäres Institut für Genetik und Funktionelle Genomforschung, Friedrich-Ludwig-Jahnstraße 15a, 17475 Greifswald; Michael Bauer, Universitätsklinikum Jena, Klinik für Anästhesiologie und Intensivtherapie, Erlanger Allee 101, 07747 Jena; Michael Kiehntopf, Universitätsklinikum Jena, Integrierte Biobank, Erlanger Allee 101, 07747 Jena; Trinad Chakraborty, Justus-Liebig-Universität Gießen, Institut für Klinische Mikrobiologie, Frankfurter Straße 107, 35392 Gießen.

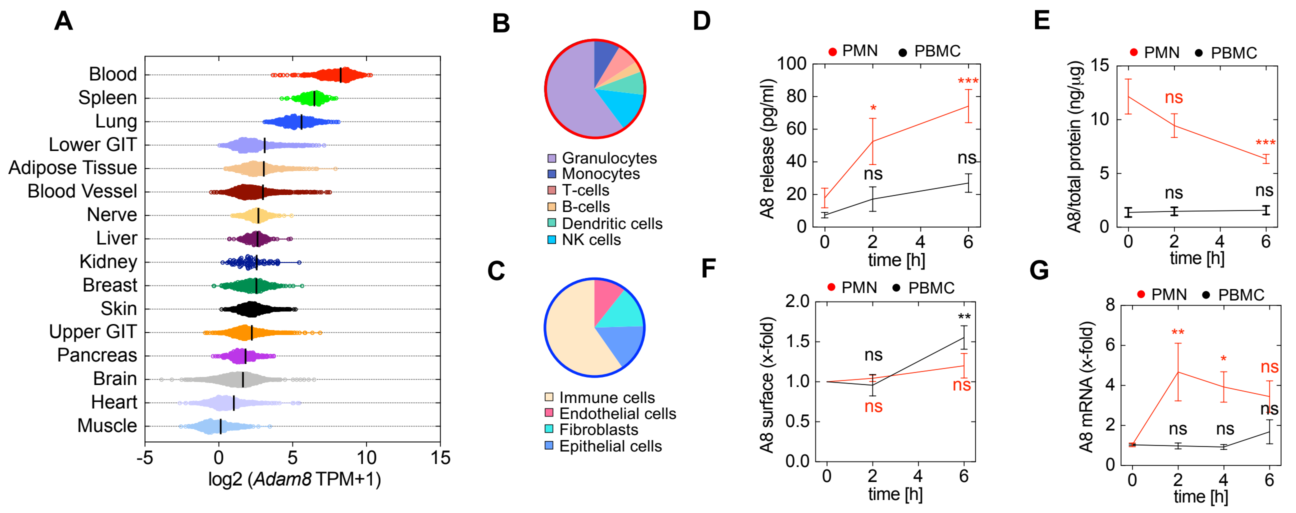

Figure S1: ADAM8 is primarily expressed and released by neutrophils during acute inflammation. **(A)** Expression pattern of *ADAM8* across healthy tissues based on the GTex human biospecimen reference database (GTEx Analysis Release V8, ENSG00000151651.15). ADAM8 transcript levels are highest in whole blood (mean transcripts per million (TPM): 304.4), followed by the spleen (mean TPM: 89.03) and the lung (mean TPM: 49.03). **(B)** Among the cells in the blood, *ADAM8* was most enriched in granulocytes. **(C)** Within normal lung tissue, *ADAM8* is primarily expressed in immune cells, including macrophages, granulocytes and lymphocytes. To compare the ADAM8 levels among the main immune effectors during inflammatory activation, we isolated human PMNs and PBMCs, and assessed **(D)** ADAM8 release, **(E)** whole cell protein, **(F)** surface and **(G)** mRNA expression over time at baseline and following stimulation with TNFα. Data are given as mean ± SEM of 4 biological replicates;*, $p < 0.05$; **, $p < 0.01$; ***, $p < 0.001$; two-way ANOVA followed by Sidak's multiple comparison test. Activation of PMNs resulted in the rapid release of soluble ADAM8 into the supernatant, with a corresponding decline in cell-associated ADAM8. In contrast, TNFα-induced ADAM8 secretion by monocytic immune cells was not significant. The surface expression of ADAM8 remained stable in PMNs, whereas PBMCs showed an increase only at 6 hours after TNFα stimulation. ADAM8 mRNA expression was induced early after stimulation in neutrophils and showed no significant change in PBMCs. Together, these data suggest that PMNs are the major source of ADAM8 following acute immune activation, while mononuclear cells exhibit a stable expression pattern in the early inflammatory phase.

Figure S1

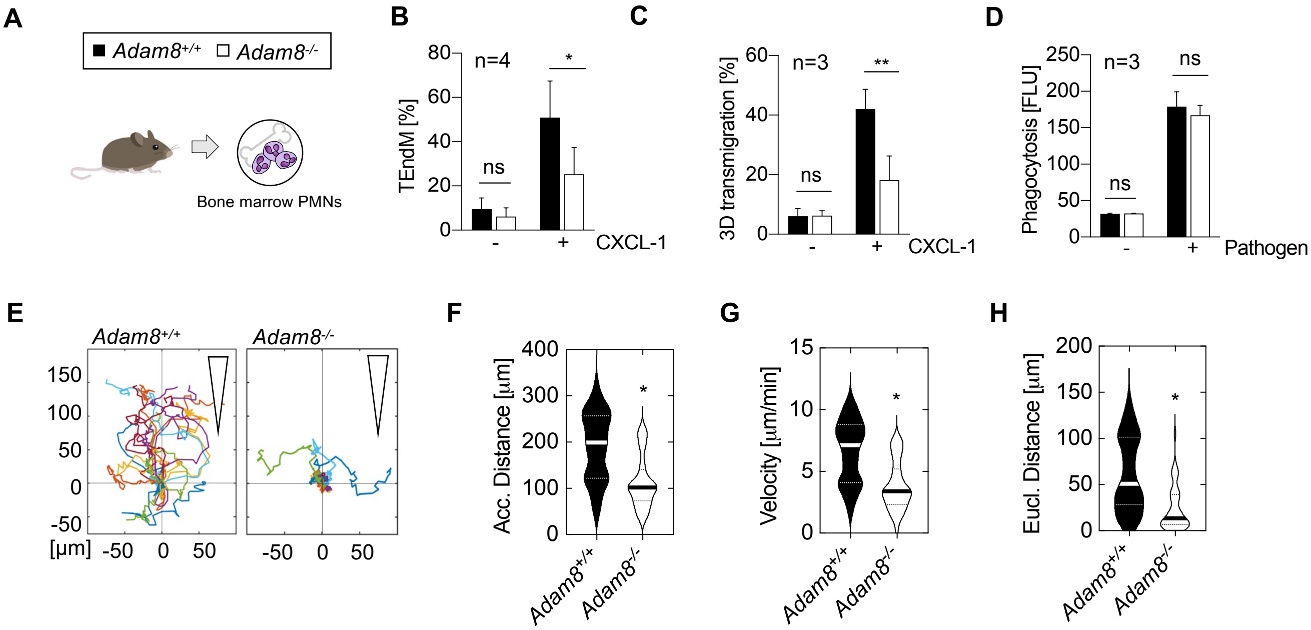

Figure S2: Functional analysis of *Adam8*$^{+/+}$ and *Adam8*$^{-/-}$ neutrophils. To investigate the role of ADAM8 in neutrophil migration, we used transwell and 3D gel-matrix based *in vitro* assays. **(A)** Bone marrow neutrophils from *Adam8* deficient mice (*Adam8*$^{-/-}$, white bars) or littermate controls (*Adam8*$^{+/+}$, black bars) were isolated and investigated *in vitro*. **(B)** Transmigration of A*dam*8$^{+/+}$ (black bars) and *Adam8*$^{-/-}$ (white bars) neutrophils across an endothelial monolayer (TEndM) or **(C)** 3D transmigration across matrigel-coated transwells stimulated by CXCL-1 (100 ng/mL). Percentage of transmigrated cells for **(B-C)** was calculated based on the number of neutrophils added to the transwell. Transmigration defects of *Adam8*$^{-/-}$ neutrophils indicate that ADAM8 is instrumental for the passage of neutrophils through tissue barriers. **(D)** Phagocytosis of pathogen particles (*E. coli*) labeled with pH-sensitive pHrodo (FLU, fluorescent units). Data for **(A-D)** are means ± SD; *, $p < 0.01$; **, $p < 0.005$; two-way ANOVA and Sidak's multiple comparison test. **(E-H)** To investigate motility in interstitial spaces, 3D chemotaxis assays on microslides (µ-Slides) were performed and the velocities of *Adam8*$^{+/+}$ and *Adam8*$^{-/-}$ neutrophils towards a CXCL-1 gradient in matrigel were analyzed. **(E)** 3D chemotactic migration (matrigel 200µg/ml) of *Adam8*$^{+/+}$ and *Adam8*$^{-/-}$ neutrophils towards a CXCL-1 (100 ng/mL) gradient using chemotaxis µ-slides. Representative trajectory plots are shown, the triangle indicates the orientation of the gradient. **(F)** Violin plots of mean accumulated distance (in µm), **(G)** velocity (µm/min) and **(H)** Euclidian distance (in µm) of *Adam8*$^{+/+}$ and *Adam8*$^{-/-}$ neutrophils. Data correspond to 3 independent experiments, 30 cells per experiment were analyzed. *, $p < 0.01$, Student's t-test.

	Mice	Venules	Diameter [µm]	$V_{centerline}$ [µm s^{-1}]	Shear rate [s^{-1}]	WBC [µl^{-1} blood]
Adam8$^{+/+}$	5	19	29 ± 4	3.8 ± 0.26	2.4 ± 0.16	4734 ± 678
Adam8$^{-/-}$	6	22	31 ± 6	3.8 ± 0.16	2.4 ± 0.10	5541 ± 935
p-value			ns (0.13)	ns (0.90)	ns (0.90)	ns (0.12)

Figure S3: Rheologic parameters of cremaster venules analyzed in Figure 1A-D.

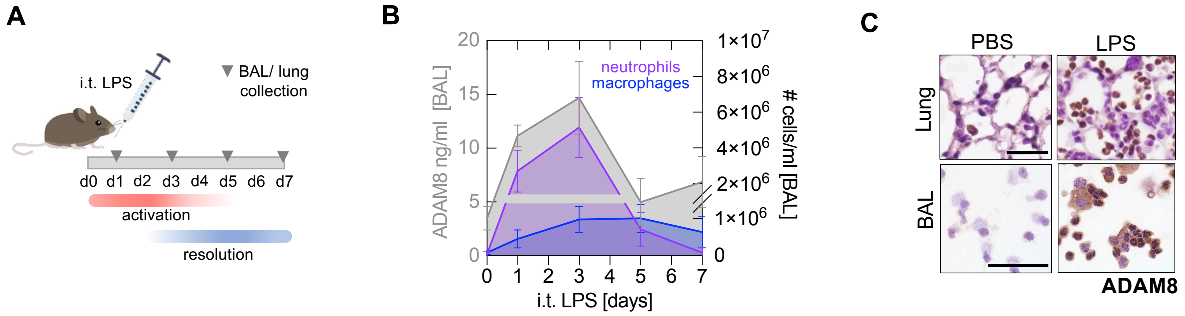

Figure S4: ADAM8 levels during LPS-induced lung inflammation are dependent on neutrophils. **(A)** To determine ADAM8 expression during acute pulmonary inflammation *in vivo*, we challenged mice with intratracheal (i.t) instillations of lipopolysaccharide (LPS) and collected BAL samples across the activation and resolution phases of inflammation. Mice were sacrificed on days (d) 1, 3, 5, and 7 after LPS challenge (grey bar, grey triangles). **(B)** Soluble ADAM8 levels (grey) were temporally associated with neutrophil accumulation (violet) in the BAL during the course of LPS-induced lung inflammation ($r=0.8236$, $p<0.0001$), whereas no relationship was detected between ADAM8 and macrophage (blue) recruitment ($r=0.1824$, $p=0.3529$), n=4-6 per timepoint. **(C)** Leukocytes recruited into the alveolar space following LPS instillation in mice are immunopositive for ADAM8, whereas the lung epithelium and endothelium lacked ADAM8 staining. Upper panel, lung tissue: lower panel, BAL cytospin. Scale bar, 25 μm.

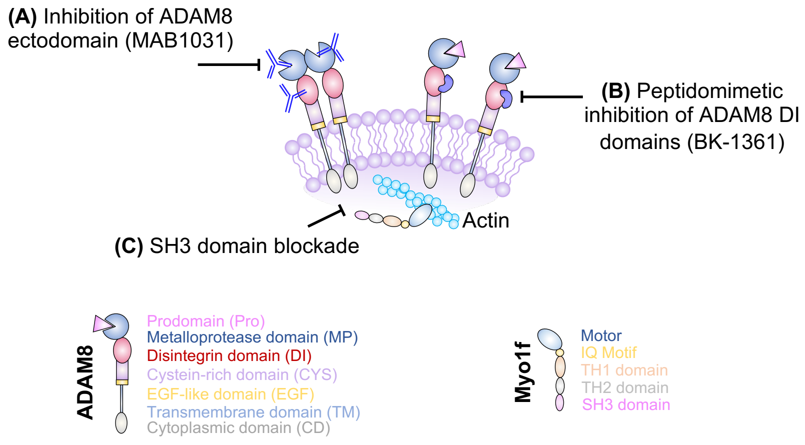

Figure S5: Schematic model of drug actions on ADAM8 and Myo1f. (A) The monoclonal antibody MAB1031 (R&D) is directed against the ectodomain of ADAM8 (aa 201-497) and has been shown to inhibit the catalytic activity of ADAM8 previously *(48)*. **(B)** The cyclic peptide BK-1361 is a small peptidomimetic that interacts with the 'KDX' motif in the integrin-binding-loop of human and mouse ADAM8, which efficiently blocks ADAM8 multimerization and subsequent auto-activation (*33*). **(C)** Blocking of SH3 domains *(49)* could prevent the intracellular interaction of ADAM8 and Myo1f. Protein domains of ADAM8 and Myo1f (lower panels).

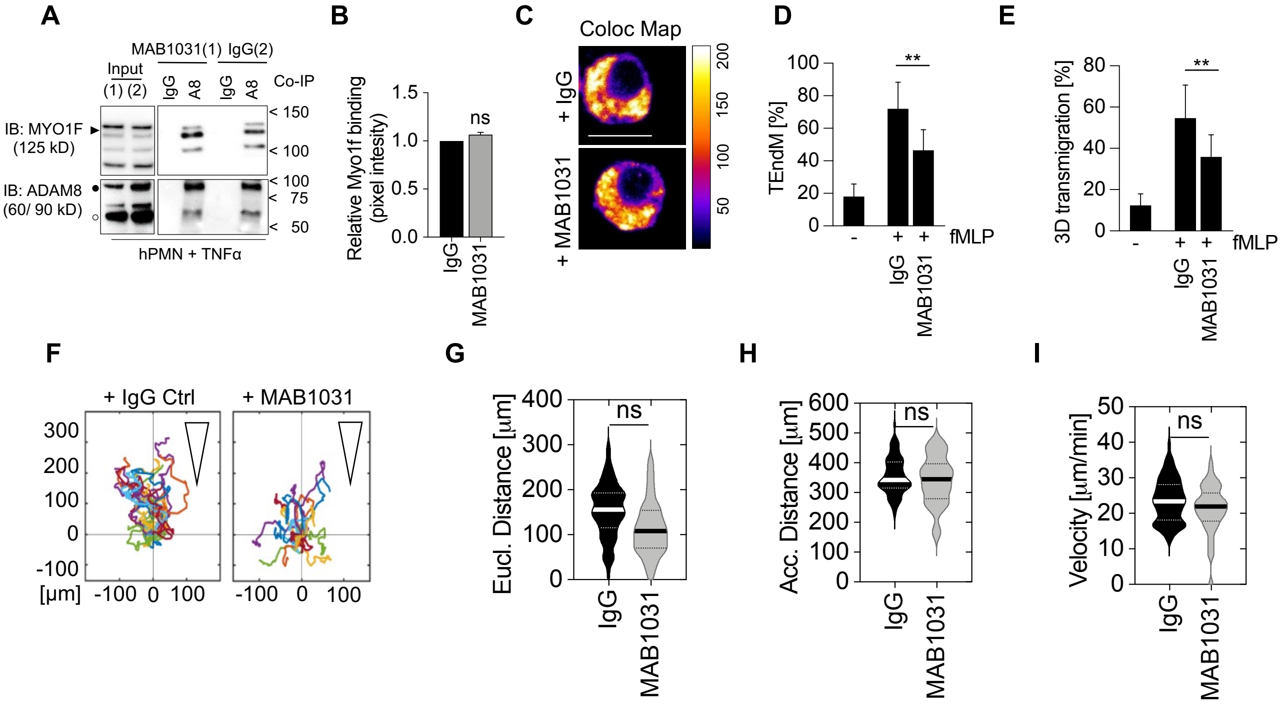

Figure S6: Effect of ADAM8 ectodomain inhibition using a monoclonal antibody. (A) Co-IP of activated human neutrophils treated with either an ADAM8 activity blocking antibody (MAB1031) or control IgG (IgG) during TNFα stimulation. **(B)** Relative pixel intensity of Myo1f co-precipitated with ADAM8 normalized to input using ImageJ Software, ns, not significant, Student's t-test. **(C)** Colocalization map (Coloc Map) of human neutrophils treated with MAB1031 or IgG as described in D. Scale bar, 10 µM. **(D)** Effect of MAB1031 on fMLP-induced transendothelial migration of human neutrophils (n=4 donors) and **(E)** migration across a 3D gel matrix (matrigel 200µg/ml), respectively (n=3 donors). Data are given as mean ± SD, stars indicate **, $p < 0.002$; Student's t-test. **(F)** 3D chemotactic migration (matrigel 200µg/ml) of human neutrophils preincubated with MAB1031 or control (IgG) toward an IL-8 (1 µg/mL) gradient using chemotaxis µ-slides. Representative trajectory plots are shown, triangles indicate orientation of the gradient. Violin plots of **(G)** mean Euclidian distance, **(H)** accumulated distance and **(I)** migration velocity of human neutrophils preincubated with MAB1031 (grey) or IgG control (black). Data correspond to 3 independent experiments, 30 cells per experiment were analyzed. ns, not significant; Student's t-test.

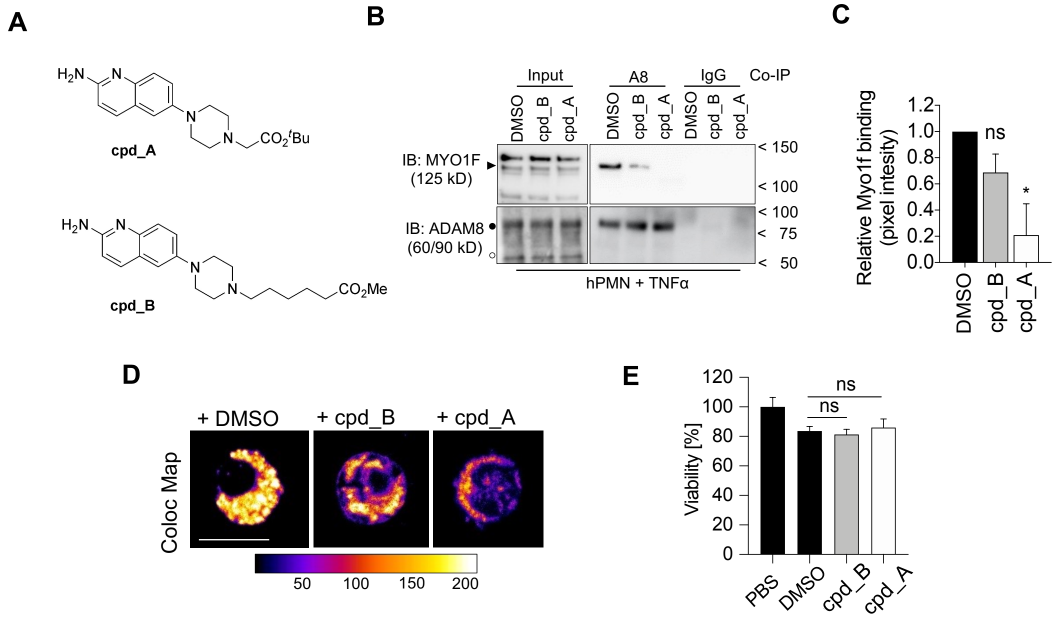

Figure S7: SH3 domain inhibition attenuates the ADAM8-Myo1f interaction. (A) Chemical structure of two inhibitors with the potential to block SH3 domains [cpd_A, compound A, cpd_B, compound B, described previously *(49)*]. **(B)** Human neutrophils were treated with 10μM cpd_A, cpd_B or DMSO as vehicle control during TNFα stimulation; representative immunoblot of ADAM8/Myo1f Co-IP is shown. **(C)** Corresponding densitometric analysis of Myo1f binding to ADAM8 normalized to input using ImageJ Software. * $p < 0.05$, ns; not significant, one-way ANOVA followed by Tukey's multiple comparison test. **(D)** Colocalization map of human neutrophils treated with SH3 inhibitors/vehicle as described in **(A)** stained for Myo1f and ADAM8. **(E)** MTT viability assay of primary human neutrophils incubated with 10μM cpd_A, cpd_B, vehicle control (DMSO) or PBS for the duration of functional assays, n=3. ns; not significant, one-way ANOVA followed by Tukey's multiple comparison test.

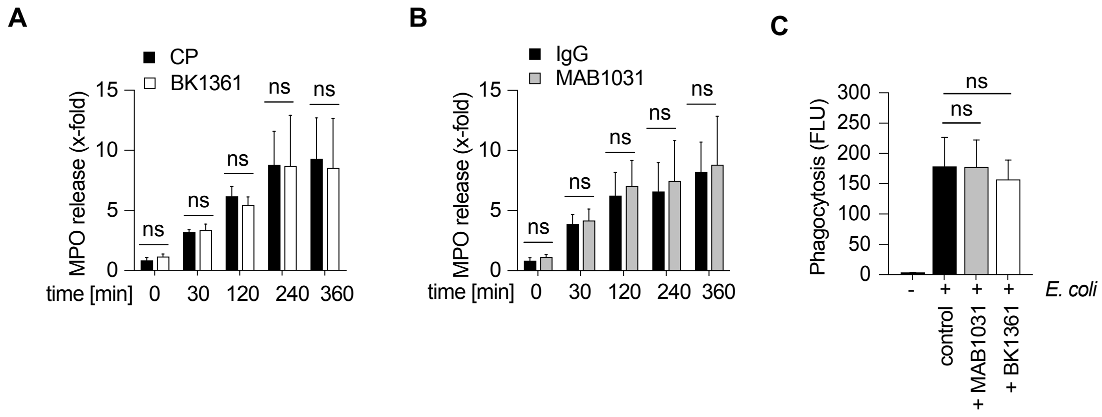

Figure S8: Effect of ADAM8 inhibition on MPO release and phagocytosis in neutrophils. MPO release by primary human neutrophils stimulated with TNFα over time quantified by ELISA. Pharmacological ADAM8 inhibition with **(A)** BK-1361 (white bars) or **(B)** MAB1031 (grey bars) was not altered compared to respective controls (black bars). n=3, ns, not significant; 2-way ANOVA followed by Sidak's multiple comparison test. **(C)** Phagocytic activity of PMNs measured by the internalization of pHrodo-conjugated *E.coli* bioparticles (FLU, fluorescent units), respectively. n=3, ns, not significant; 1-way ANOVA followed by Sidak's multiple comparison test.

Figure S8

A

peptide #	cleavage sequence	homologous cleavage site
005	LAQAPhe(homo)RSK	Pro-TNFα
008	PChaGC(Me)HAK	-
010	SPLAQAVRSSK	Pro-TNFα
011	GPLGMRGK	-
013	HGDQMAQKSK	CD23
014	EHADLLAVVAK	Pro-TGFα
052	APFEMSAK	-

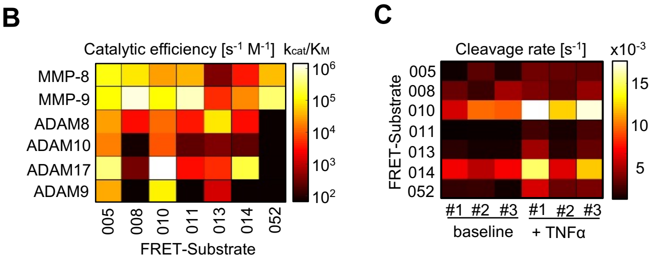

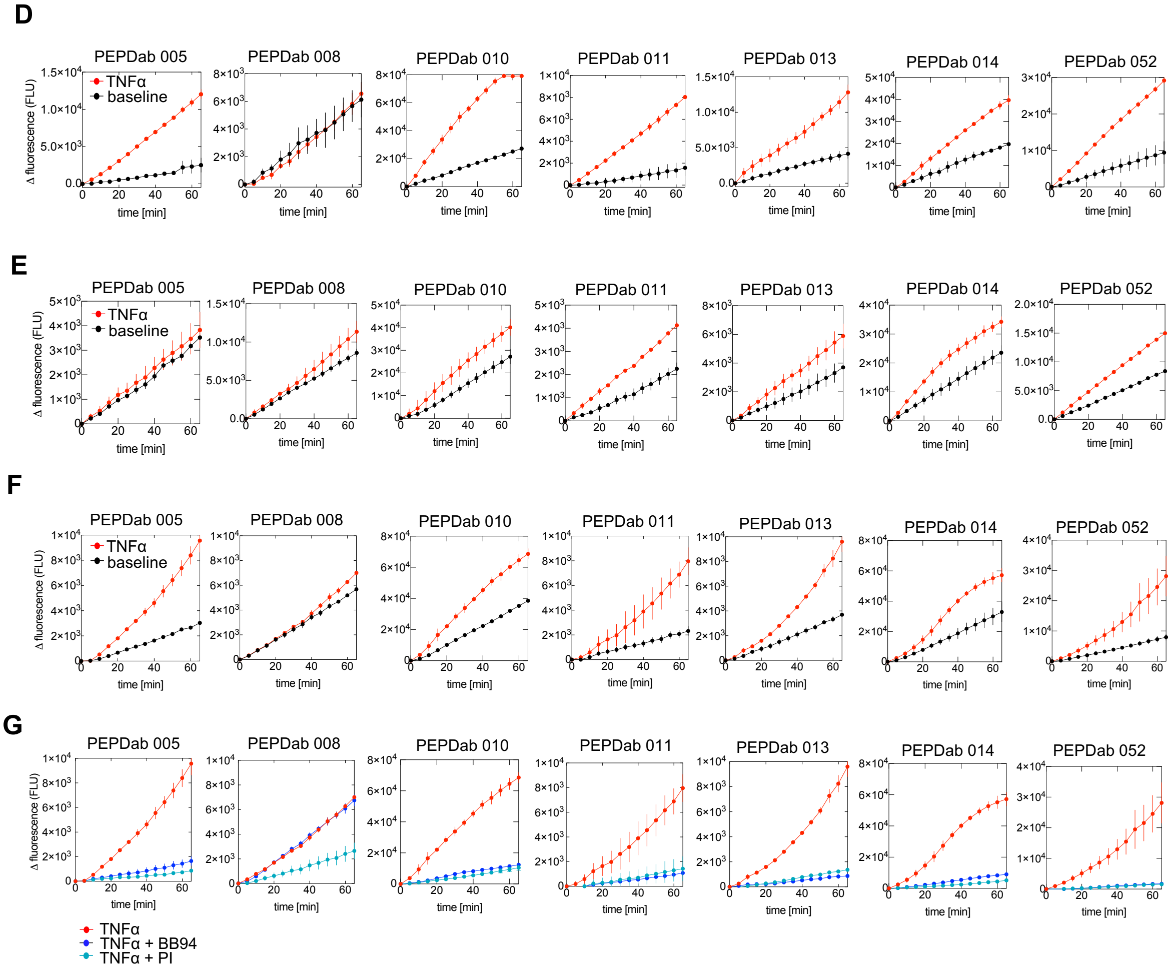

Figure S9: Proteolytic profiling of human neutrophils. (A) Cleavage sequences of fluorescent substrates and, if applicable, homologous cleavage sites in endogenous substrates. **(B)** Standard table of catalytic efficiencies of recombinant MMP-8, MMP-9, ADAM8, ADAM9, ADAM10 and ADAM17 for FRET-based peptide substrates 005, 008, 010, 011, 013, 014 and 052, as described previously (Miller et al., 2011). **(C)** Substrate cleavage patterns of isolated human neutrophils obtained from three donors at baseline and after stimulation with TNFα (20ng/ml) were calculated from (D-F) using a non-linear kinetic model. **(D-F)** Time-lapse fluorimetry (fluorescence change of time) of unstimulated (black) and stimulated (red) human neutrophils using a panel of seven FRET-based peptide substrates (PEPDab 005, 008, 010, 013, 014 and 052, Biozyme Inc., Apex, NC) to deduce a profile of specific MMP and ADAM proteolytic activities. **(G)** Proteolytic activities were reduced by ~90% using cOmplete Protease Inhibitor (green) with broad inhibitory specificity against serine, cysteine, and metalloproteases, and blocked by ~70-90% using the broad-spectrum metalloproteinase inhibitor batimastat BB-94 (blue). Representative time-lapse data are plotted for TNFα stimulated neutrophils from (F) and respective inhibitors treatments.

Figure S9

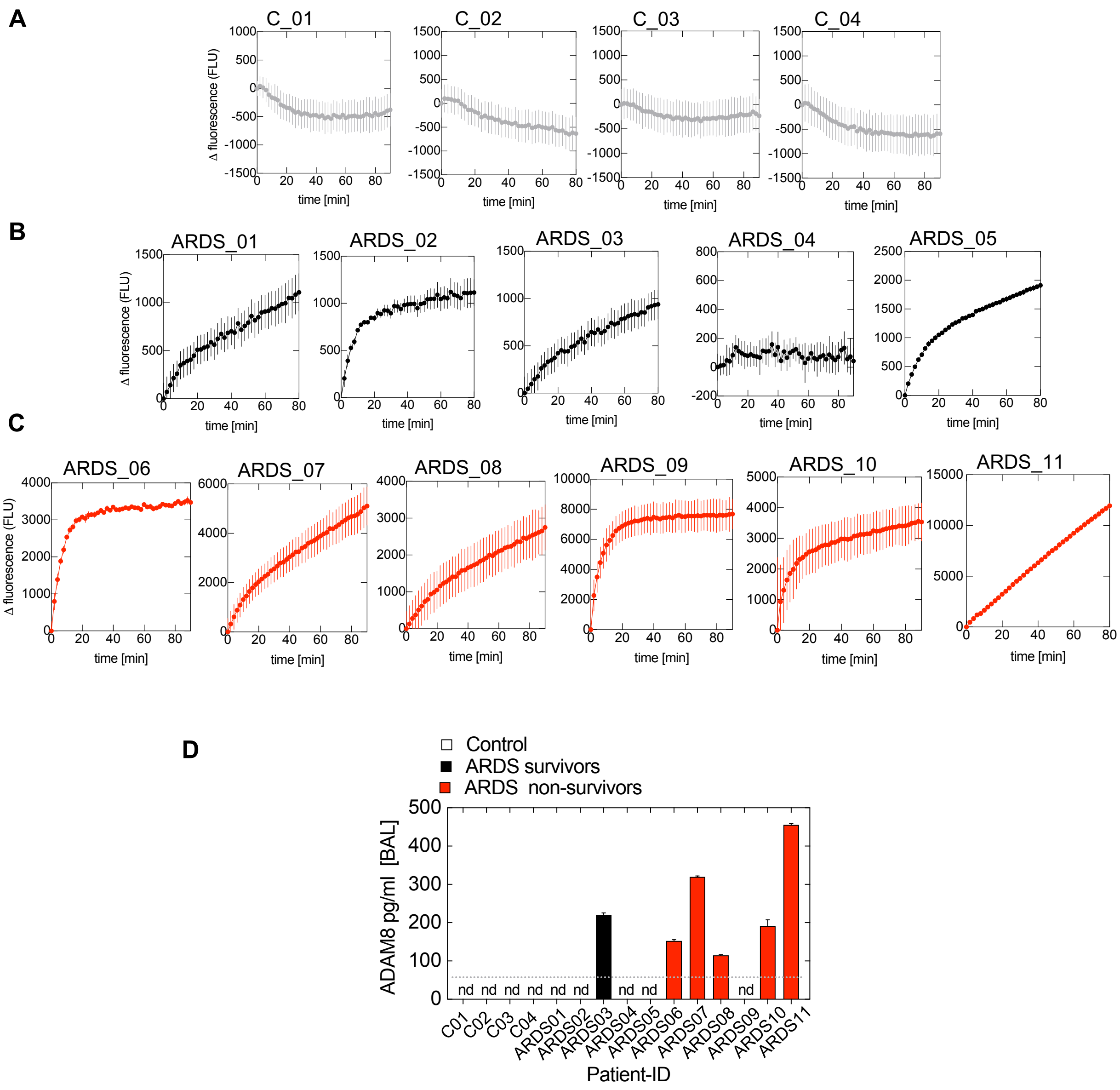

Figure S10: PEPDab013 cleavage and ADAM8 protein levels in lung fluids of ARDS patients and healthy controls. Time-lapse fluorimetry (fluorescence change over time) of **(A)** control BAL (grey), **(B)** BAL of ARDS survivors (black) and **(C)** BAL of ARDS non-survivors (red) using the moderately ADAM8 specific substrate PEPDab 013. **(D)** Detection of soluble ADAM8 in BAL samples of control (white), ARDS survivors (black) and ARDS non-survivors using ELISA, respectively. Dotted grey line, sensitivity cut-off 50pg/ml; nd, not detectable.

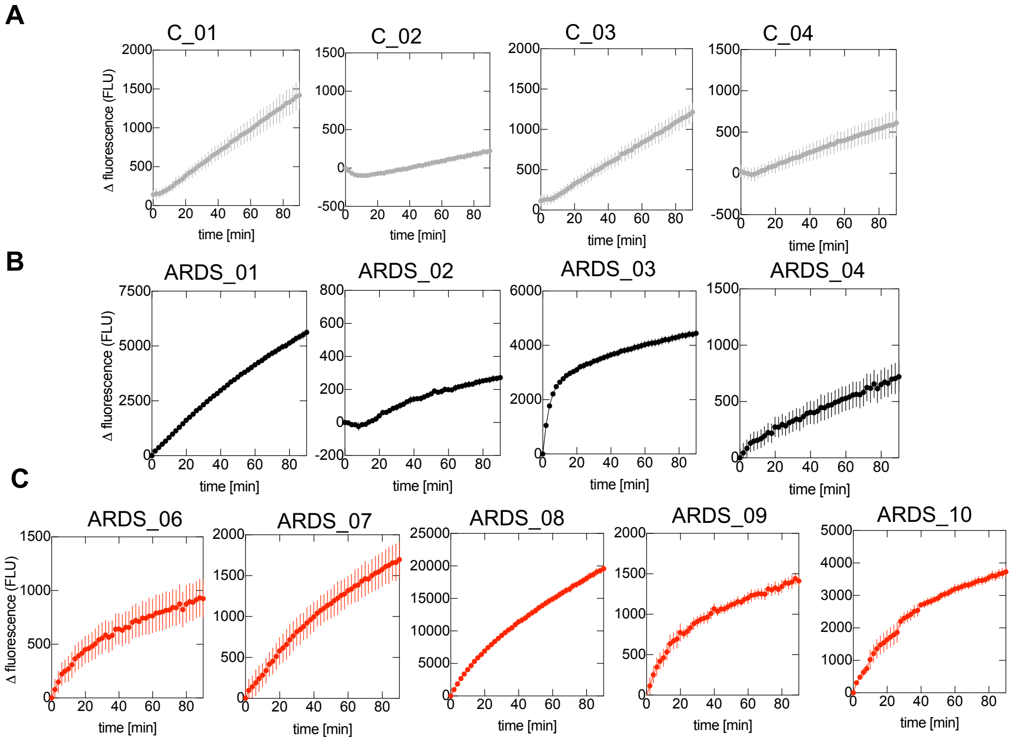

Figure S11: PEPDab014 cleavage in lung fluids of ARDS patients and healthy controls. Time-lapse fluorimetry (fluorescence change over time) of **(A)** control BAL (grey), **(B)** BAL of ARDS survivors (black) and **(C)** BAL of ARDS non-survivors (red) using the moderately ADAM17 specific substrate PEPDab014.

Figure S11

Lung tissue *(P.a., Adam8$^{-/-}$)*

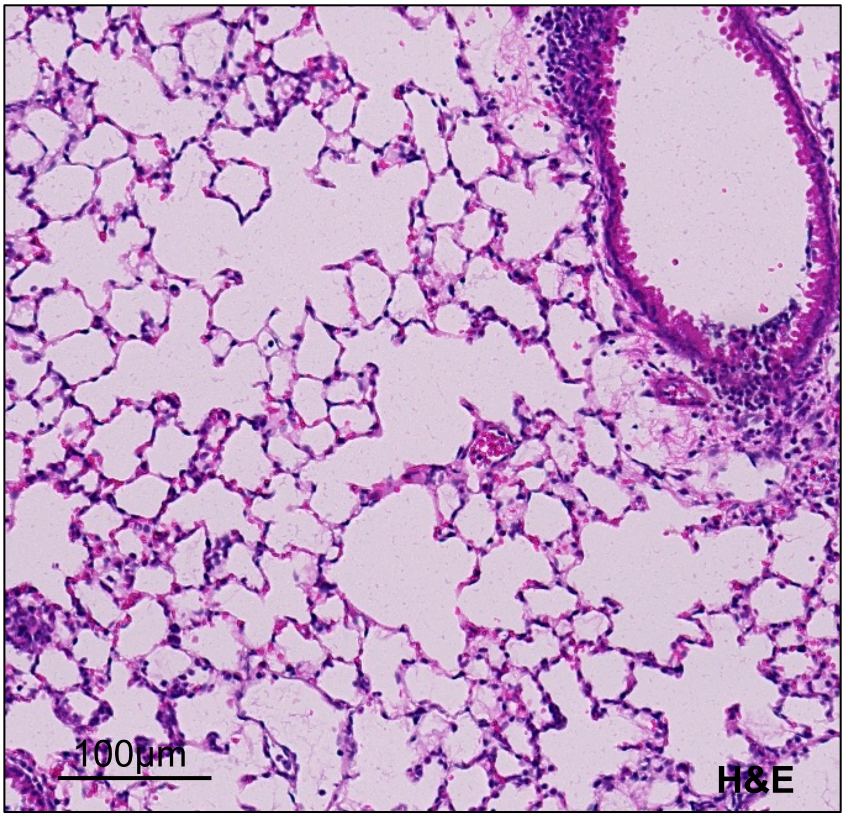

Lung tissue *(P.a.,Adam8$^{+/+}$)*

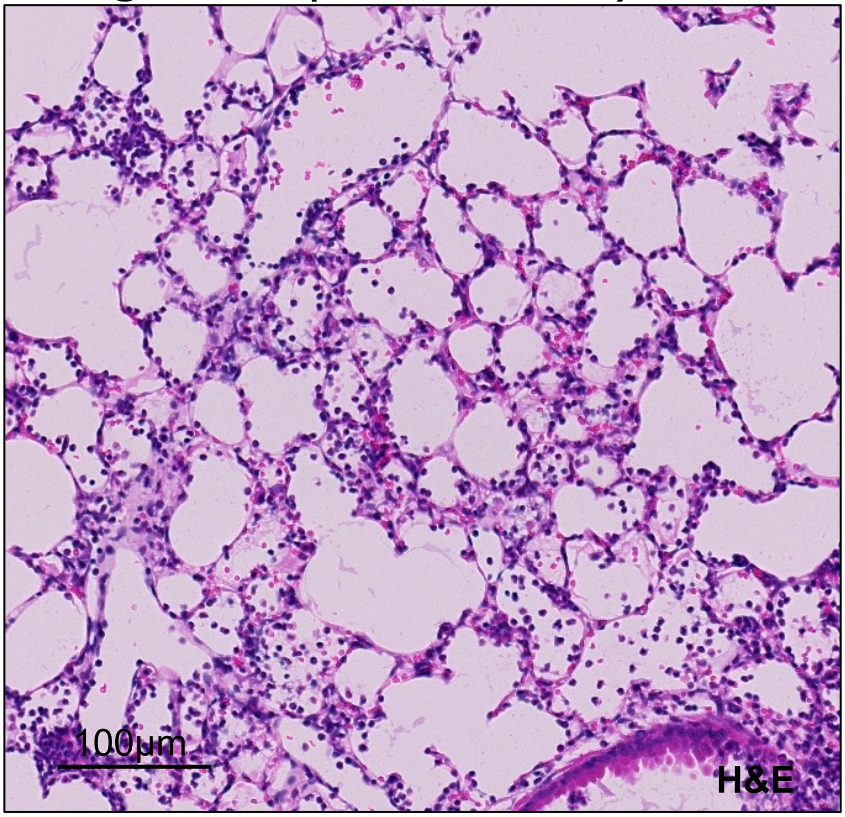

Supplemental Figure 12. Lower power field magnification of Figure 3G.

Lung tissue (*P.a.* + BK1361)

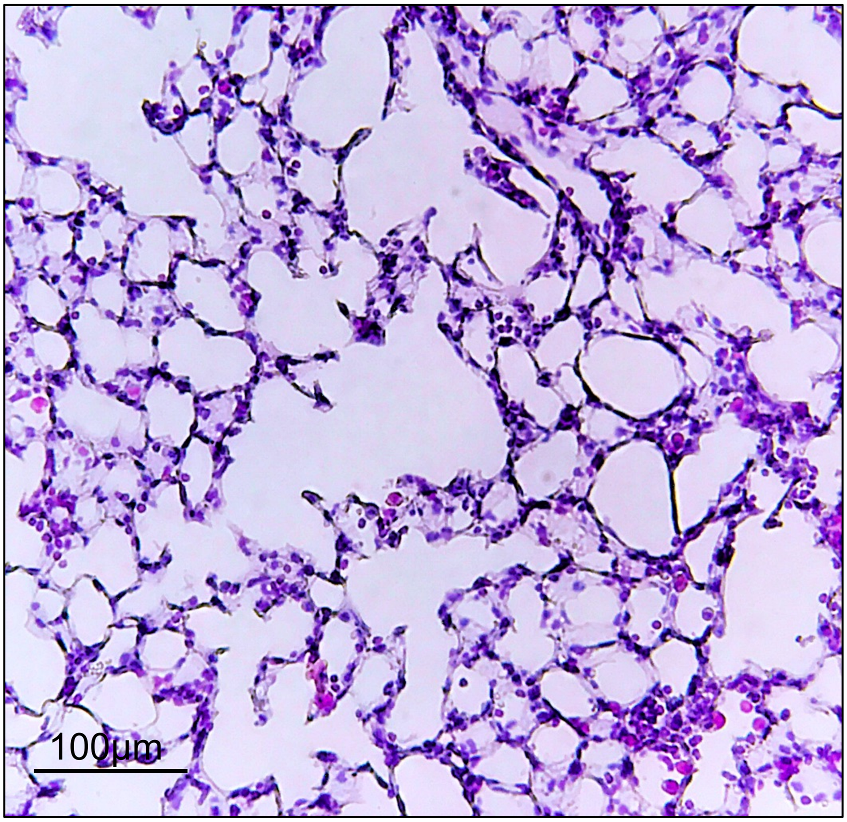

Lung tissue (*P.a.* + CP)

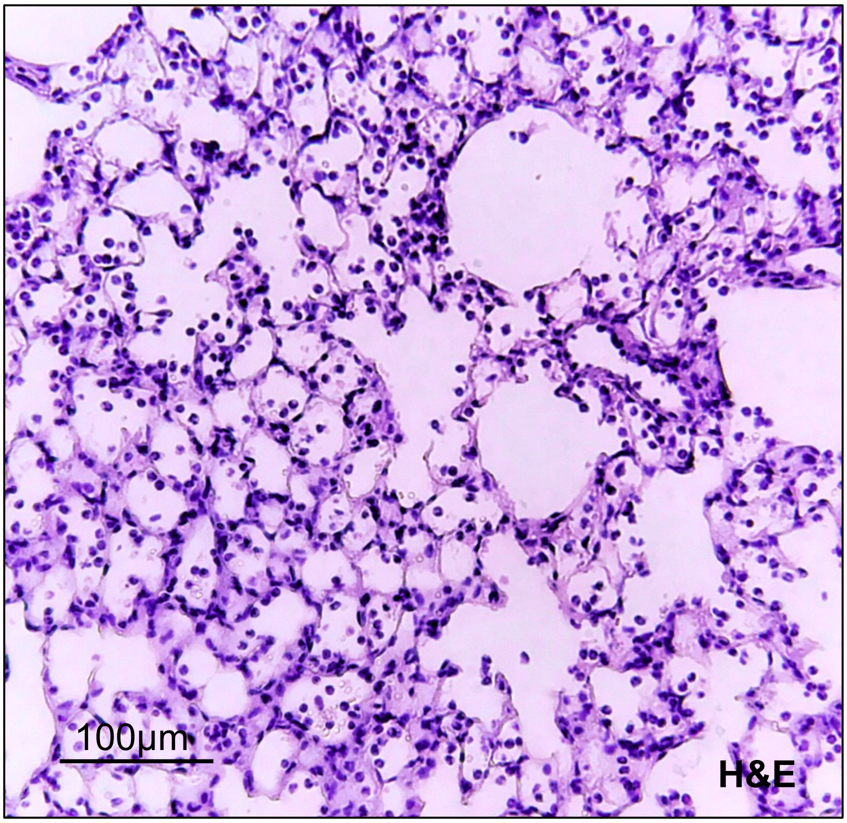

Lower powerfield magnification of Figure 3R.

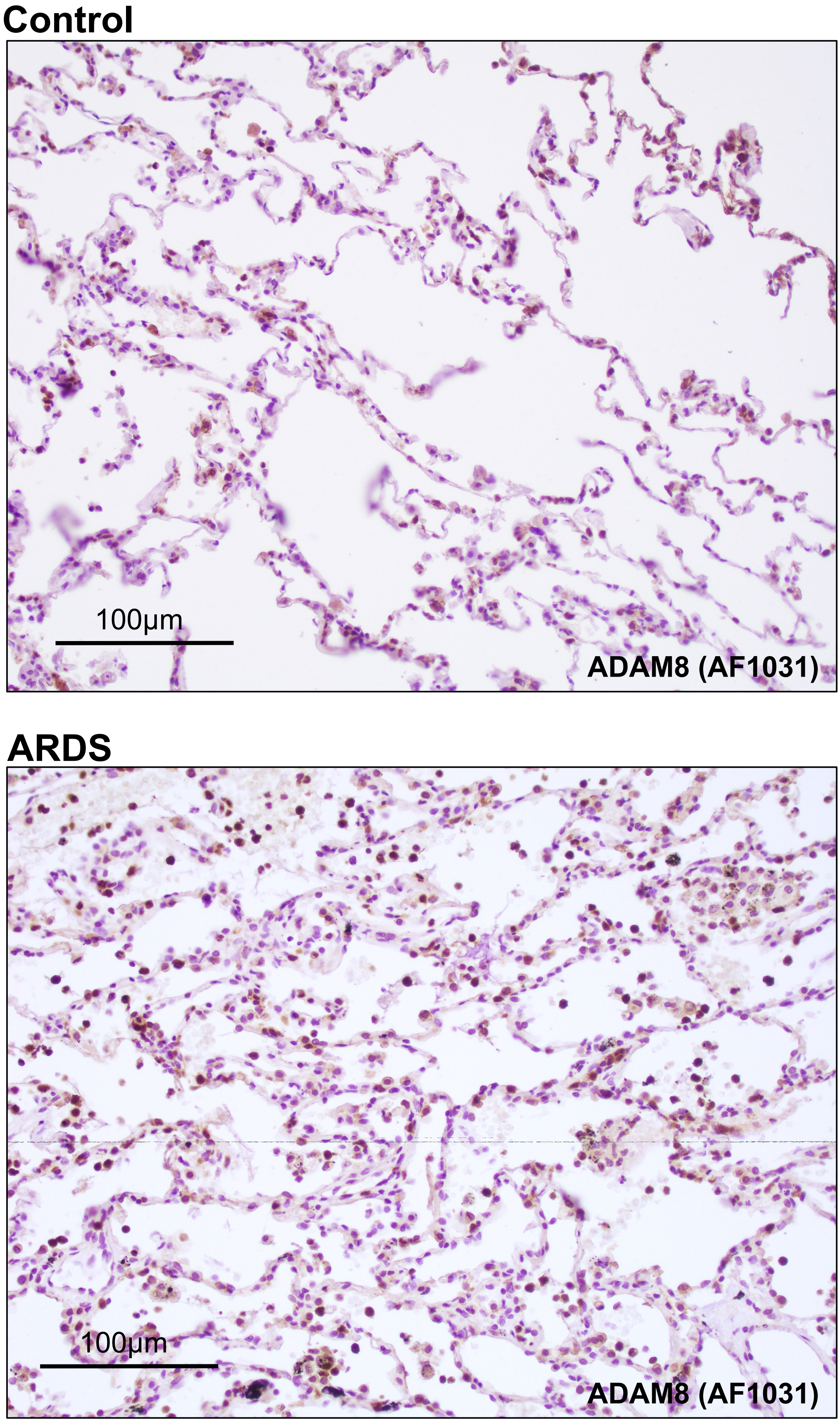

Lower powerfield magnification of Figure 4c.

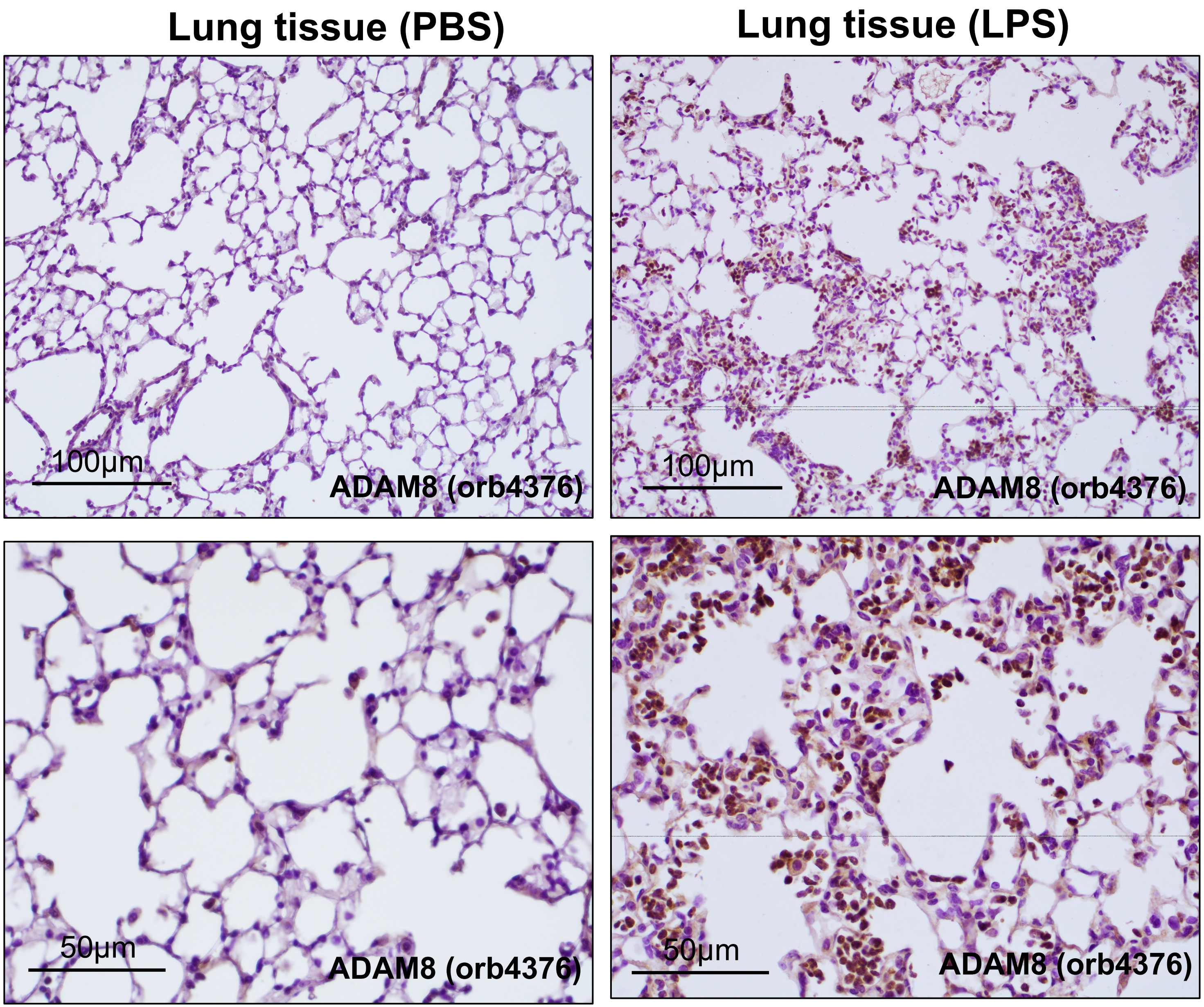

Lower powerfield magnification of Figure S4c.

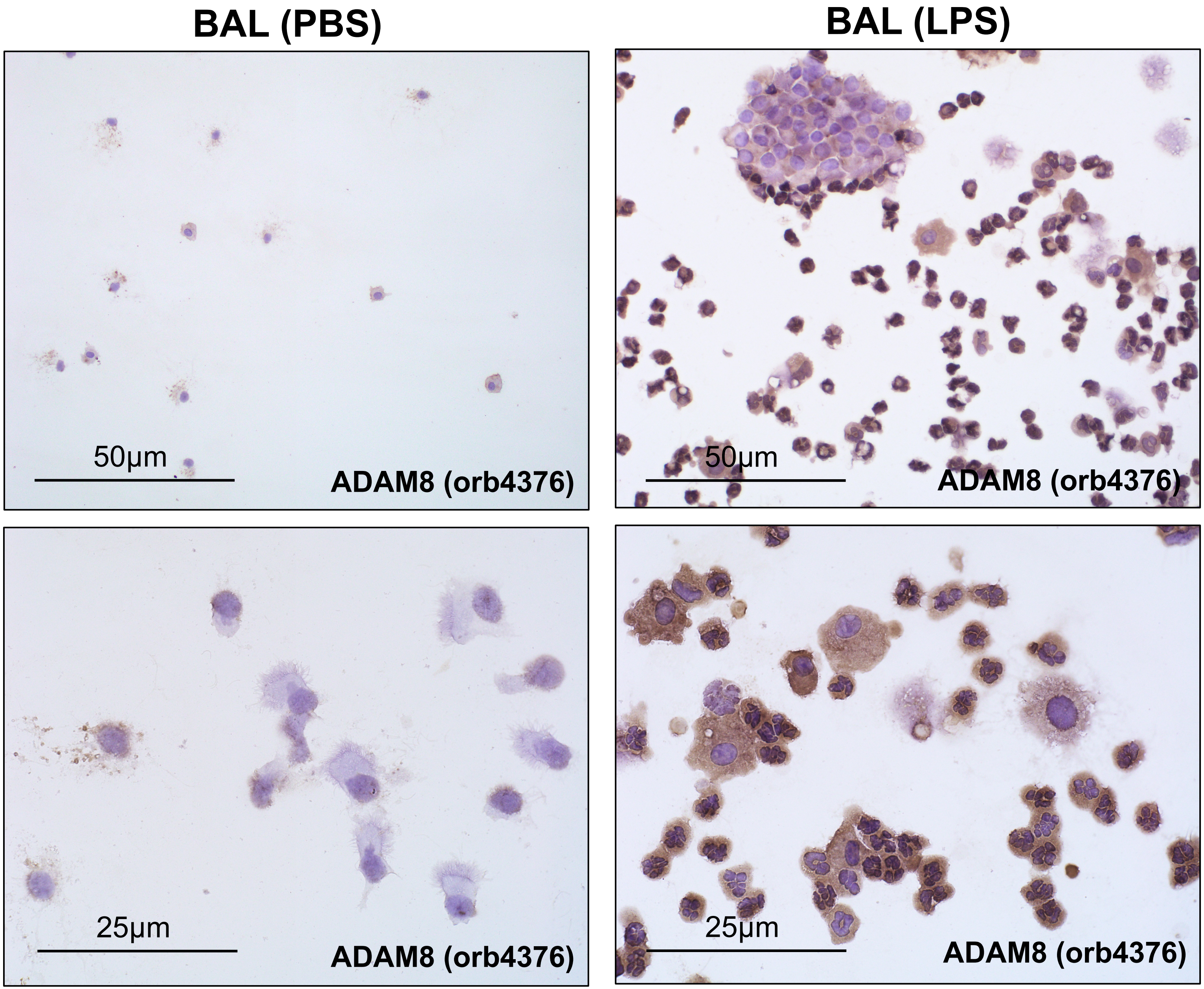

Lower powerfield magnification of Figure S4c.

Mouse Clinical Assessment Score for Sepsis (M-CASS)				
**Score**	**1**	**2**	**3**	**4**
**Fur Aspect**	Normal fur	Slightly ruffled fur	Ruffled fur	Ruffled fur and piloerection
**Activity**	Normal	Reduced	Only when provoked	Little or none with provocation
**Posture**	Normal	Hunched, moving freely	Hunched, strained or stiff movement	Hunched, little or no movement
**Behavior**	Normal	Slow	Abnormal when disturbed or provoked	Abnormal, no relocation
**Chest Movements**	Normal	Mild dyspnea	Moderate dyspnea	Severe dyspnea
**Eyelids**	Normal, open	Opened when disturbed	Partially closed, even when disturbed	Mostly or completely closed, even when provoked

Table S1: Surrogate endpoint scoring systems for assessing disease severity in mice. Mouse Clinical Assessment Score for Sepsis (M-CASS).

Act ADAM8 [nM]	Sex	Age	ARDS etiology	BAL cells	Outcome
0.830	F	61	Pneumonia (bacterial)	78% PMN 14% Macrophages 8% Lymphocytes	non-survivor
0.634	M	55	Pneumonia (viral)	62% PMN 11% Macrophages 7% Lymphocytes	non-survivor
0.516	M	47	Pneumonia (bacterial, viral)	82% PMN 13% Macrophages 5% Lymphocytes	non-survivor
0.472	F	52	Pneumonia (bacterial, viral, fungal)	87% PMN 11% Macrophages 7% Lymphocytes	non-survivor
0.403	F	51	Pneumonia (viral)	85% PMN 4% Macrophages 9% Lymphocytes	non-survivor
0.399	F	56	Pneumonia (bacterial, viral)	73% PMN 26% Macrophages 1% Lymphocytes	non-survivor
0.368	F	30	Pneumonia (bacterial, viral)	71% PMN 19 % Macrophages 10% Lymphocytes	survivor
0.340	M	40	Pneumonia (bacterial, viral)	N/A	survivor
0.298	M	50	Pneumonia (bacterial, viral) pre-existing lung fibrosis	51% PMN 42% Macrophages 7% Lymphocytes	survivor, required lung transplantation
0.211	M	53	Pneumonia (bacterial, viral)	19% PMN 49% Macrophages 30% Lymphocytes	survivor
0.177	F	24	Healthy	N/A	control
0.139	M	20	Pneumonia (bacterial, viral)	71% PMN 19% Macrophages 7% Lymphocytes	survivor
0.139	M	27	Healthy	N/A	control
0.095	M	23	Healthy	N/A	control
0.077	F	23	Healthy	N/A	control

Table S2: Summary of clinical data of patients with ARDS due to pneumonia.

Act ADAM8 [nM]	PGD	Sex	Age	Transplant indication	BAL cells	MV [d]	ICU stay [d]
1.310	Severe	F	63	Non-IPF ILD	87% PMN<br>13% Macrophages<br>0% Lymphocytes	33	42
1.15	Severe	F	21	Chronic transplant rejection	0% PMN<br>100% Macrophages<br>0% Lymphocytes	N/A	28
0.897	Severe	M	68	IPF	90% PMN<br>6% Macrophages<br>4% Lymphocytes	2	8
0.698	Severe	M	67	Non-IPF ILD	52% PMN<br>48% Macrophages<br>0% Lymphocytes	46	50
0.560	Mild	F	54	Non-IPF ILD	55% PMN<br>45% Macrophages<br>0% Lymphocytes	5	13
0.558	Severe	M	70	IPF	N/A	N/A	N/A
0.497	Severe	M	66	NSIP	21% PMN<br>78% Macrophages<br>1% Lymphocytes	41	31
0.447	Severe	F	32	CTD-ILD	72% PMN<br>27% Macrophages<br>1% Lymphocytes	40	40
0.444	Severe	M	61	COPD	47% PMN<br>53% Macrophages<br>0% Lymphocytes	28	35
0.332	Severe	F	60	Non-IPF ILD	74% PMN<br>21% Macrophages<br>5% Lymphocytes	57	48
0.324	Mild	M	63	CTD-ILD	N/A	2	4
0.282	Mild		51	Non-IPF ILD	48% PMN<br>49% Macrophages<br>3% Lymphocytes	3	6
0.278	Severe	M	66	IPF	75% PMN<br>23% Macrophages<br>2% Lymphocytes	4	6
0.264	Mild	F	50	CF	30% PMN<br>68% Macrophages<br>1% Lymphocytes	1	3
0.248	Severe	F	33	PAH (idiopathic)	N/A	N/A	18
0.214	Mild	M	69	IPF	N/A	1	5
0.195	Severe	F	74	Non-IPF ILD	81% PMN<br>17% Macrophages<br>2% Lymphocytes	1	5
0.194	Severe	M	68	IPF	88% PMN<br>11% Macrophages<br>1% Lymphocytes	N/A	14
0.153	Mild	F	41	CF	54% PMN<br>44% Macrophages<br>2% Lymphocytes	1	4
0.15	Mild	F	65	IPF	71% PMN<br>24% Macrophages<br>5% Lymphocytes	1	5
0.145	Mild	M	57	Non-IPF ILD	20% PMN<br>75% Macrophages<br>5% Lymphocytes	1	7
0.141	Severe	M	33	NSIP	79% PMN<br>21% Macrophages<br>3% Lymphocytes	2	7
0.141	Mild	M	70	COPD	N/A	0	7
0.121	Mild	F	31	CF	55% PMN<br>43% Macrophages<br>4% Lymphocytes	4	8

Table S3

0.114	Mild	M	70	Hypersensitivity Pneumonitis	53% PMN<br>45% Macrophages<br>2% Lymphocytes	2	4
0.085	Mild	M	69	IPF	80% PMN<br>18% Macrophages<br>2% Lymphocytes	4	6
0.0797	Severe	M	49	CTD-ILD	77% PMN<br>21% Macrophages<br>3% Lymphocytes	3	7
0.0751	Mild	M	68	Sarcoidosis	N/A	2	5
0.067	Severe	M	48	Chronic transplant rejection	86% PMN<br>13% Macrophages<br>1% Lymphocytes	2	12
0.0653	Mild	F	61	COPD	N/A	N/A	5
0.047	Mild	M	49	CF	N/A	1	5
0.019	Mild	M	42	Non-IPF ILD	N/A	21	N/A

Table S3: Summary of clinical data of patients with ARDS due to Primary Graft Dysfunction (PGD). IPF, idiopathic fibrosis; ILD, Interstitial Lung Disease; NSIP, Non-specific interstial pneumonia; CTD; Connective Tissue Disease; COPD, Chronic obstructive pulmonary disease; CF, Cystic fibrosis; PAH, Pulmonary arterial hypertension.

Supplemental Table 4. Table of Key Resources

REAGENT/RESOURCE	SOURCE	IDENTIFIER
Antibodies		
eFluor450-conjugated Mouse CD11c (Clone: 418)	ThermoFisher	Cat # 48-0114-82, RRID: AB_464940
FITC-conjugated Mouse CD11b (Clone: M1/70)	ThermoFisher	Cat # 14-0112-82, RRID: AB_467108
PE-conjugated Mouse CD86 (Clone: B7-2)	ThermoFisher	Cat # 12-0862-82, RRID: AB_465768
APC-conjugated Mouse Ly6G (Clone: 1A8)	ThermoFisher	Cat # 17-9668-82, RRID: AB_2573307
PerCP-Cy5.5-conjugated Mouse CD45 (Clone: 30-F11)	BioLegend	Cat # 103131, RRID: AB_893344
PE-Cy7-conjugated Mouse F4/80 (Clone: BM8)	ThermoFisher	Cat # 25-4801-82, RRID: AB_469653
Human ADAM8 ectodomain (Clone: 143303)	R&D Systems	Cat # MAB1031, RRID: AB_2305036
Human ADAM8 Polyclonal Ectodomain Antibody	R&D Systems	Cat # AF1031, RRID: AB_354549
Human ADAM8 Polyclonal Cytoplasmic Domain Antibody	LSBio	Cat # LS-B4068-50, RRID: AB_10718869
Mouse IgG2B Isotype Control (Clone: 20116)	R&D Systems	Cat # MAB004, RRID: AB_357346
Human Myo1f Polyclonal Antibody	Biorbyt	Cat # orb221550
Anti-Goat IgG H&L (Alexa Fluor® 568)	Abcam	Cat # ab175474, RRID: AB_2636995
Anti-Rabbit IgG H&L (Alexa Fluor® 488)	Abcam	Cat # ab150077, RRID: AB_2630356
Anti-Rabbit IgG, HRP-linked Antibody	Cell Signaling	Cat # 7074, RRID: AB_2099233
Anti-Mouse IgG, HRP-linked Antibody	Cell Signaling	Cat # 7076, RRID: AB_330924
Anti-Goat IgG, HRP-linked Antibody	ThermoFisher	Cat # PA1-28664, RRID: AB_10990162
Mouse Myeloperoxidase Polyclonal	R&D Systems	Cat # AF3667, RRID: AB_2250866
Mouse ADAM8 Polyclonal	R&D Systems	Cat # orb4376, RRID: AB_10948754
Mouse Ly6G (Clone: 1A8)	Bio X Cell	Cat # BE0075-1, RRID: AB_1107721
Mouse S100A8	R&D Systems	Cat # AF3059, RRID: AB_2184254
Anti-Rat IgG, Cy3 linked Antibody	Jackson ImmunoResearch	Cat # 712-165-153, RRID: AB_2340667
Anti-Goat IgG, AF488 linked Antibody	Jackson ImmunoResearch	Cat # 705-545-003, RRID: AB_2340428
Hoechst 33342 Solution	ThermoFisher	Cat # 62249
DAPI (4',6-Diamidino-2-Phenylindole, Dihydrochloride)	ThermoFisher	Cat # D1306
Fixable Yellow Dead Cell Stain	ThermoFisher	Cat # L34967
Bacterial and Virus Strains		
Pseudomonas aeruginosa (Strain PA103)	ATCC	ATCC 29260
Biological Samples		
Lung fluids of patients with ARDS due to pneumonia	Universities of Giessen and Marburg Lung Center, Pneumonia Biorepository	UGMLC/DLZ, Az 58/15 / Az 87/12
Lung fluids of healthy volunteers	Universities of Giessen and Marburg Lung Center	UGMLC/DLZ, Az 58/15 / Az 87/12
Lung fluids of patients with ARDS due to PGD	University of California, San Francisco, Lung Transplant Biorepository	IRB # 13-10738
Lung tissue from ARDS and non-ARDS patients	University of Texas Health and Science Center, Houston	IRB # HSC-MS-08-0354
Chemicals, Peptides, and Recombinant Proteins		
N-Formyl-Met-Leu-Phe (fMLP)	Sigma	Cat # F3506
Lipopolysaccharide (O111:B4)	Sigma	Cat # L4391
Mouse TNFα	GenScript	Cat # Z03333
Mouse Cxcl-1/KC	GenScript	Cat # Z02899
Human TNFα	GenScript	Cat # Z01001
Human IL-8	GenScript	Cat # Z03061
Batimastat (BB-94)	Sigma	Cat # SML0041

cOmplete Protease Inhibitor	Roche	Cat # 11697498001
TRIzol Reagent	ThermoFisher	Cat # 15596026
Matrigel	Corning	Cat # 356237
**Critical Commercial Assays**		
Mouse Myeloperoxidase ELISA	R&D Systems	Cat # DY3667
Mouse Cxcl-1 ELISA	R&D Systems	Cat # DY453
Mouse TNFα ELISA	R&D Systems	Cat # DY410
Mouse ADAM8 ELISA	Biomatik	Cat # EKU02052
Mouse Albumin ELISA	Bethyl Laboratories	Cat # E90-134
Human ADAM8 ELISA	R&D Systems	Cat # DY1031
Human Myeloperoxidase ELISA	R&D Systems	Cat # DY3174
Pierce BCA Protein Assay Kit	ThermoFisher	Cat # 23225
Dynabeads Antibody Coupling Kit	ThermoFisher	Cat # 14311D
VECTASTAIN Elite ABC-HRP Kit, Peroxidase (goat IgG)	Vector Laboratories	Cat # PK-6105
VECTASTAIN Elite ABC-HRP Kit, Peroxidase (Rabbit IgG)	Vector Laboratories	Cat # PK-6101
DAB Substrate Kit	Vector Laboratories	Cat # SK-4100
pHrodo Green *E. coli* BioParticles	ThermoFisher	Cat # P35366
High Capacity cDNA Reverse Transcription Kit	ThermoFisher	Cat # 4368814
Power SYBR Green PCR Master Mix	ThermoFisher	Cat # 4368706
TaqMan Fast Advanced Master Mix	ThermoFisher	Cat # 4444556
μ-Slide Chemotaxis	Ibidi	Cat # 80326
6.5mm Transwell Insert, 8μm Pore Size	Corning	Cat # 3422
**Deposited Data**		
GTEx Analysis Release V8 (dbGaP Accession phs000424.v8.p2), ENSG00000151651.15	GTexPortal	https://gtexportal.org/home/datasets
**Experimental Models: Cell Lines**		
Mouse: Brain-derived Endothelial Cells (b.End3), p5	ATCC	ATCC CRL-2299
Human: Microvascular Endothelial Cells (HMEC-1), p9	ATCC	ATCC CRL-3243
Human: Leukocyte Cell Line (HL-60), p0	ATCC	ATCC CRL-240, LOT: 64048671
**Experimental Models: Organisms/Strains**		
Mouse: (WT) C57BL/6	Jackson Laboratory	Cat# 000664; RRID: IMSR_JAX:000664
Mouse: (Adam8 ko), *Adam8*$^{-/-}$	A.J.P. Docherty, UCB, Slough, UK	N/A
Mouse: B6.Cg-Tg(S100A8-cre,-EGFP)1Ilw/J	Jackson Laboratory	Cat# 021614; RRID: IMSR_JAX:021614
Mouse: D2.129(Cg)-Gt(ROSA)26Sortm4(ACTB-tdTomato,-EGFP)Luo/SjJ	Jackson Laboratory	Cat# 026862, RRID: IMSR_JAX:026862
**Oligonucleotides**		
Human ADAM8 KiCqStart Primers	Sigma	Cat # H_ADAM8_1
Human ACTB KiCqStart Primers	Sigma	Cat # H_ACTB_1
Human GAPDH KiCqStart Primers	Sigma	Cat # H_GAPDH_1
Mouse IL1β TaqMan Gene Expression Assay (FAM)	Thermo Fisher	Cat # 4331182, Mm00434228_m1
Mouse TNFα TaqMan Gene Expression Assay (FAM)	Thermo Fisher	Cat # 4331182, Mm00443258_m1
Mouse GAPDH TaqMan Gene Expression Assay (FAM)	Thermo Fisher	Cat # 4331182, Mm99999915_g1
Eukaryotic 18S TaqMan Gene Expression Assay (FAM)	ThermoFisher	Cat # 4331182, Hs03003631_g1
**Software and Algorithms**		
ImageJ Software	NIH	https://imagej.nih.gov/ij/
FlowJo Software	TreeStar	https://www.flowjo.com/
Imaris	Oxford Instruments	https://imaris.oxinst.com/
GraphPad Prism V8 Software	GraphPad	https://www.graphpad.com/
Matlab 2020b Software	MathWorks	https://www.mathworks.com
